# The top 100 most cited articles on axon regeneration from 2003 to 2023: a bibliometric analysis

**DOI:** 10.3389/fnins.2024.1410988

**Published:** 2024-06-26

**Authors:** Saijilafu, Ling-Chen Ye, Jing-Yu Zhang, Ren-Jie Xu

**Affiliations:** ^1^Key Laboratory of Novel Targets and Drug Study for Neural Repair of Zhejiang Province, School of Medicine, Hangzhou City University, Hangzhou, China; ^2^Department of Orthopaedics, Suzhou Municipal Hospital, The Affiliated Suzhou Hospital of Nanjing Medical University, Suzhou, China

**Keywords:** axon regeneration, bibliometric analysis, Web of Science Core Collection, VOSviewer, CiteSpace

## Abstract

**Objective:**

In this study, we used a bibliometric and visual analysis to evaluate the characteristics of the 100 most cited articles on axon regeneration.

**Methods:**

The 100 most cited papers on axon regeneration published between 2003 and 2023 were identified by searching the Web of Science Core Collection database. The extracted data included the title, author, keywords, journal, publication year, country, and institution. A bibliometric analysis was subsequently undertaken.

**Results:**

The examined set of 100 papers collectively accumulated a total of 39,548 citations. The number of citations for each of the top 100 articles ranged from 215 to 1,604, with a median value of 326. The author with the most contributions to this collection was He, Zhigang, having authored eight papers. Most articles originated in the United States (*n* = 72), while Harvard University was the institution with the most cited manuscripts (*n* = 19). Keyword analysis unveiled several research hotspots, such as chondroitin sulfate proteoglycan, alternative activation, exosome, Schwann cells, axonal protein synthesis, electrical stimulation, therapeutic factors, and remyelination. Examination of keywords in the articles indicated that the most recent prominent keyword was “local delivery.”

**Conclusion:**

This study offers bibliometric insights into axon regeneration, underscoring that the United States is a prominent leader in this field. Our analysis highlights the growing relevance of local delivery systems in axon regeneration. Although these systems have shown promise in preclinical models, challenges associated with long-term optimization, agent selection, and clinical translation remain. Nevertheless, the continued development of local delivery technologies represents a promising pathway for achieving axon regeneration; however, additional research is essential to fully realize their potential and thereby enhance patient outcomes.

## 1 Introduction

A neuron is composed of four distinct components, namely, the soma, dendrites, axon, and presynaptic terminals. These highly specialized cellular structures constitute the basis for interneuron communication. The primary function of an axon is to transmit the excitatory signals generated by the soma to other neurons or effectors. Axon regrowth after injury can restore the connectivity between neurons and target cells, thereby reinstating the integrity of neural circuits. Consequently, axon regeneration plays a crucial role in functional recovery following neural damage.

While axons readily regenerate in the peripheral nervous system (PNS) following injury, spontaneous axon regrowth in the central nervous system (CNS) is significantly limited. Regeneration in the adult mammalian CNS, including the spinal cord, is challenging due to limited plasticity and the presence of inhibitory factors resulting from myelin degradation. Recent advancements in spinal cord injury research have revealed that the CNS possesses a greater inherent regenerative capacity than previously believed (Barnabé-Heider and Frisén, 2008; Okano and Yamanaka, 2014); however, this capacity does not match that observed in the PNS.

Numerous articles have been published on axon regeneration, rendering it challenging for researchers to identify the most influential papers, research trends, and future directions in this field. Bibliometric analysis pertains to the statistical evaluation of published papers or books. Performing an in-depth bibliometric analysis of the most cited papers on axon regeneration would make a significant contribution to the understanding of the future development directions in this discipline. Accordingly, the objective of this study was to identify the 100 most cited papers on axon regeneration and analyze their bibliometric characteristics, thereby discerning the research trends and future directions in this field.

## 2 Materials and methods

### 2.1 Search strategy and data extraction

**Figure 1:**
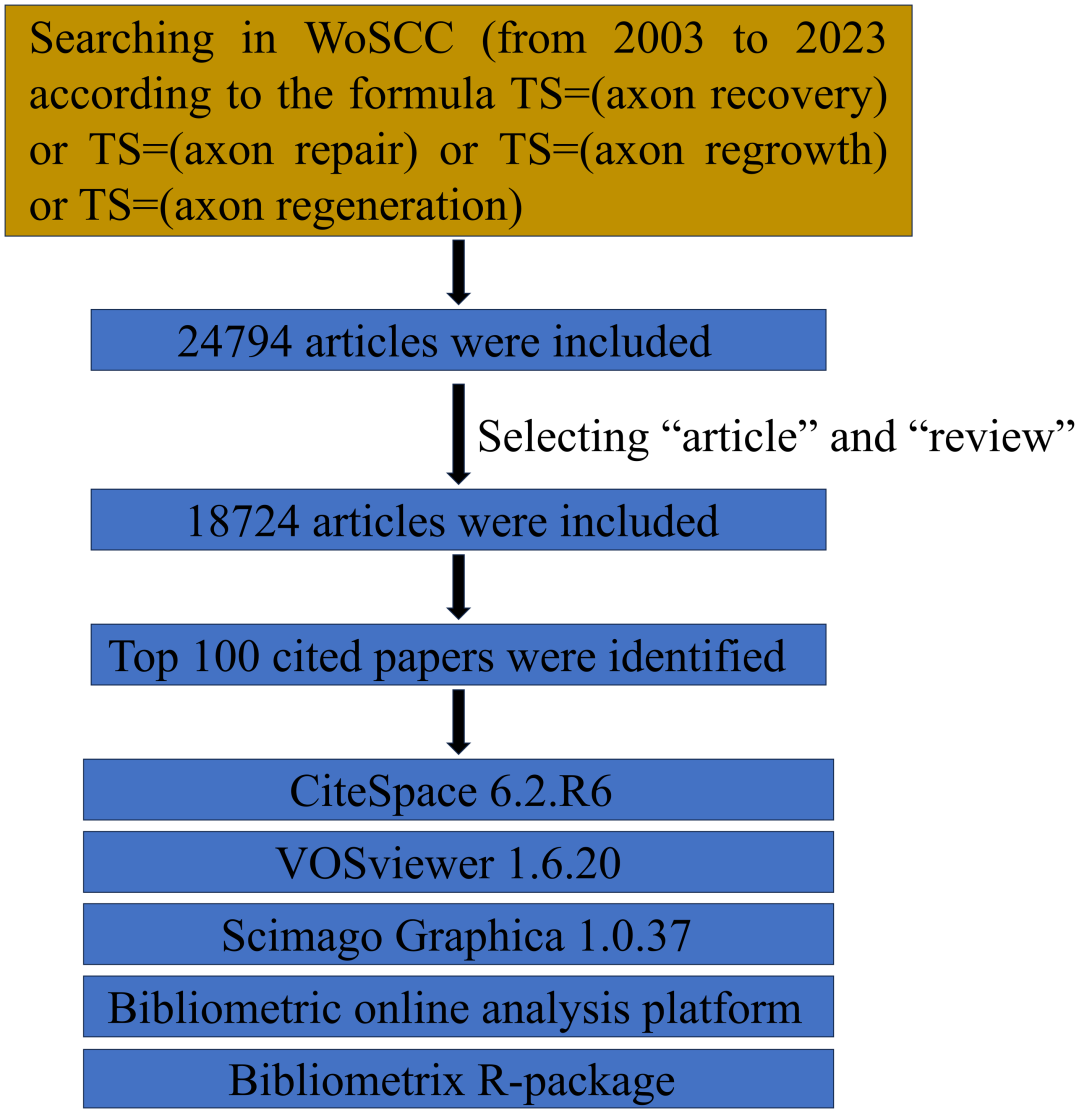
Document screening flow chart.

The data for this study were extracted from the Science Citation Index Expanded (SCI-EXPANDED) within the Clarivate Analytics Web of Science Core Collection (WoSCC). The WoSCC is a highly esteemed global database that provides comprehensive information for use with bibliometric software and is a popular choice for bibliometric analysis. TS = (axon recovery) or TS = (axon repair) or TS = (axon regrowth) or TS = (axon regeneration) was used to search for relevant articles. A systematic search was performed within the WoSCC for literature published between January 1900 and December 2023. Document retrieval and recording were concluded on December 31, 2023, to prevent possible bias resulting from subsequent database updates. Only complete original articles and reviews were considered for bibliometric analysis, regardless of the language. For each article, two researchers separately screened the title, abstract, and document type. If necessary, the researchers perused the full article for a more comprehensive evaluation of whether to include it in the analysis. The records of the 100 most influential publications were obtained from WoSCC in the “Full Record and Cited References” format and saved as.txt files (Figure 1). The impact factor of each journal was derived from the most recent (2022) Journal Citation Reports.

### 2.2 Data analysis and visualization

Descriptive statistical analysis and diagram generation were performed using Microsoft Excel 2019. An online analytic platform^1^ was used to identify collaboration networks among authors and institutions. VOSviewer, a robust bibliometric tool developed by Van Eck and Waltman (van Eck and Waltman, 2010), was employed for the construction of networks involving keywords, authors, and more. Scimago Graphica software (Hassan-Montero et al., 2022) was used to create a world map illustrating the number of countries cited. Additionally, the downloaded data were integrated into CiteSpace (Chen, 2004) to construct author networks, identify keywords with the highest burst strength, and illustrate the relationship between time and keyword bursts. CiteSpace was also used to generate an institutional cooperation network, an author co-authorship network, and a co-occurrence network. In the network map rendered by CiteSpace, different elements, such as institutions, authors, and keywords, are represented by various nodes. The thickness of the lines connecting the nodes signifies the strength of the co-authorship and co-occurrence links. In the co-occurrence analysis, the correlation of items was determined based on the number of documents in which they co-occur, with different clusters denoted by different colors. The “bibliometrix” R package was employed for bibliometric analysis of the relevant literature. Bibliometrix allows gaining rapid insights into the seminal works, prominent figures, and future trends in the field and aids in organizing the pertinent literature and presenting the results in a visual format. One notable feature of bibliometrix is its ability to generate Sankey diagrams, also known as Sankey energy balance diagrams. These diagrams depict data flows, with the width of the branches proportional to the magnitude of the flow. They function as effective tools for visually analyzing data.

## 3 Results

### 3.1 Analysis of publications and citations

**Figure 2:**
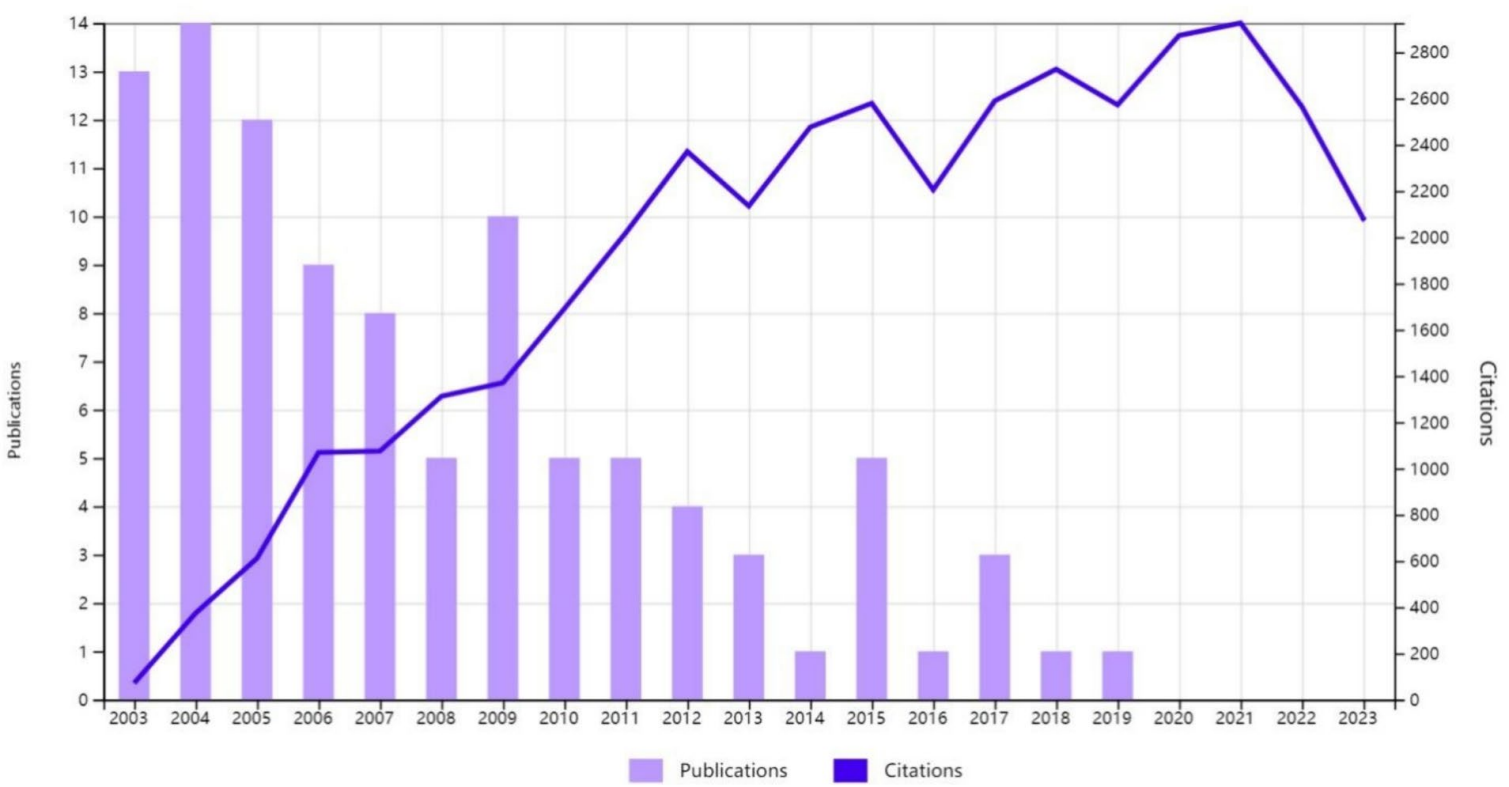
The yearly number of publications and citations for the most cited publications on axon regeneration from 2003 to 2023.

All the 100 most cited studies were published between 2003 and 2019 (Figure 2). Table 1 provides detailed information on the 100 most influential publications. The top 100 articles garnered between 215 and 1,604 citations, with a median of 326 and an average of 395.48 citations per article. Focusing on the top three studies, the most cited article (Kigerl et al., 2009) (“Identification of Two Distinct Macrophage Subsets with Divergent Effects Causing either Neurotoxicity or Regeneration in the Injured Mouse Spinal Cord”) was published in the Journal of Neuroscience in 2009 and was cited 1,604 times. The second most cited paper (Park et al., 2008) (“Promoting Axon Regeneration in the Adult CNS by Modulation of the PTEN/mTOR Pathway”) was published in Science in 2008 and received 1,187 citations. The third most cited paper (Anderson et al., 2016), titled “Astrocyte scar formation aids central nervous system axon regeneration,” was published in 2016 in Nature and received 1,139 citations. Earlier publications had an advantage in terms of the total citation count. However, when ranked according to average citations per year, some later publications were found to have a greater impact. For instance, although the above-mentioned article (Anderson et al., 2016) “Astrocyte scar formation aids central nervous system axon regeneration,” published in 2016, ranked third for total citations, it ranked first for average number of citations per year (142.38).

**Table 1:** The 100 most-cited articles in axon generation.

Number	First author	Publication year	Title	Journal	Total citations
1	Kigerl et al. (2009)	2009	Identification of Two Distinct Macrophage Subsets with Divergent Effects Causing either Neurotoxicity or Regeneration in the Injured Mouse Spinal Cord	Journal of Neuroscience	1,604
2	Park et al. (2008)	2008	Promoting Axon Regeneration in the Adult CNS by Modulation of the PTEN/mTOR Pathway	Science	1,187
3	Anderson et al. (2016)	2016	Astrocyte scar formation aids central nervous system axon regeneration	Nature	1,139
4	Liu et al. (2010)	2010	PTEN deletion enhances the regenerative ability of adult corticospinal neurons	Nature Neuroscience	689
5	Lu et al. (2003)	2003	Neural stem cells constitutively secrete neurotrophic factors and promote extensive host axonal growth after spinal cord injury	Experimental Neurology	685
6	Xin et al. (2013)	2013	Systemic administration of exosomes released from mesenchymal stromal cells promote functional recovery and neurovascular plasticity after stroke in rats	Journal of Cerebral Blood Flow and Metabolism	674
7	Mi et al. (2004)	2004	LINGO-1 is a component of the Nogo-66 receptor/p75 signaling complex	Nature Neuroscience	660
8	Lu et al. (2012)	2012	Long-Distance Growth and Connectivity of Neural Stem Cells after Severe Spinal Cord Injury	Cell	645
9	Ellis-Behnke et al. (2006)	2006	Nano neuro knitting: Peptide nanofiber scaffold for brain repair and axon regeneration with functional return of vision	Proceedings of the National Academy of Sciences of the United States of America	643
10	Schnell et al. (2007)	2007	Guidance of glial cell. Migration and axonal growth on electrospun nanofibers of poly-ε-caprolactone and a collagen/poly-ε-caprolactone blend	Biomaterials	595
11	Pearse et al. (2004)	2004	cAMP and Schwann cells promote axonal growth and functional recovery after spinal cord injury	Nature Medicine	579
12	Lee et al. (2009)	2009	Polypyrrole-coated electrospun PLGA nanofibers for neural tissue applications	Biomaterials	564
13	Tysseling-Mattiace et al. (2008)	2008	Self-assembling nanofibers inhibit glial scar formation and promote axon elongation after spinal cord injury	Journal of Neuroscience	559
14	Cattin et al. (2015)	2015	Macrophage-Induced Blood Vessels Guide Schwann Cell-Mediated Regeneration of Peripheral Nerves	Cell	532
15	Moore et al. (2009)	2009	KLF Family Members Regulate Intrinsic Axon Regeneration Ability	Science	531
16	Fournier et al. (2003)	2003	Rho kinase inhibition enhances axonal regeneration in the injured CNS	Journal of Neuroscience	531
17	Sun et al. (2011)	2011	Sustained axon regeneration induced by co-deletion of PTEN and SOCS3	Nature	521
18	Xin et al. (2013)	2013	MiR-133b Promotes Neural Plasticity and Functional Recovery After Treatment of Stroke with Multipotent Mesenchymal Stromal Cells in Rats Via Transfer of Exosome-Enriched Extracellular Particles	Stem Cells	514
19	Shen et al. (2009)	2009	PTPσ Is a Receptor for Chondroitin Sulfate Proteoglycan, an Inhibitor of Neural Regeneration	Science	502
20	Crigler et al. (2006)	2006	Human mesenchymal stem cell subpopulations express a variety of neuro-regulatory molecules and promote neuronal cell survival and neuritogenesis	Experimental Neurology	499
21	Karimi-Abdolrezaee et al. (2006)	2006	Delayed transplantation of adult neural precursor cells promotes remyelination and functional neurological recovery after spinal cord injury	Journal of Neuroscience	484
22	Gensel and Zhang (2015)	2015	Macrophage activation and its role in repair and pathology after spinal cord injury	Brain Research	480
23	Kingham et al. (2007)	2007	Adipose-derived stem cells differentiate into a Schwann cell phenotype and promote neurite outgrowth *in vitro*	Experimental Neurology	476
24	Hellal et al. (2011)	2011	Microtubule Stabilization Reduces Scarring and Causes Axon Regeneration After Spinal Cord Injury	Science	443
25	Yin et al. (2003)	2003	Macrophage-derived factors stimulate optic nerve regeneration	Journal of Neuroscience	435
26	Koh et al. (2008)	2008	Enhancement of neurite outgrowth using nano-structured scaffolds coupled with laminin	Biomaterials	421
27	Kerschensteiner et al. (2005)	2005	*In vivo* imaging of axonal degeneration and regeneration in the injured spinal cord	Nature Medicine	421
28	Stirling et al. (2004)	2004	Minocycline treatment reduces delayed oligodendrocyte death, attenuates axonal dieback, and improves functional outcome after spinal cord injury	Journal of Neuroscience	401
29	Sakai et al. (2011)	2012	Human dental pulp-derived stem cells promote locomotor recovery after complete transection of the rat spinal cord by multiple neuro-regenerative mechanisms	Journal of Clinical Investigation	399
30	Huang et al. (2011)	2011	Retinoid X receptor gamma signaling accelerates CNS remyelination	Nature Neuroscience	397
31	Yin et al. (2006)	2006	Oncomodulin is a macrophage-derived signal for axon regeneration in retinal ganglion cells	Nature Neuroscience	397
32	Nori et al. (2011)	2011	Grafted human-induced pluripotent stem-cell-derived neurospheres promote motor functional recovery after spinal cord injury in mice	Proceedings of the National Academy of Sciences of the United States of America	394
33	Atwal et al. (2008)	2008	PirB is a Functional Receptor for Myelin Inhibitors of Axonal Regeneration	Science	392
34	Koffler et al. (2019)	2019	Biomimetic 3D-printed scaffolds for spinal cord injury repair	Nature Medicine	380
35	Fouad et al. (2005)	2005	Combining Schwann cell bridges and olfactory-ensheathing glia grafts with chondroitinase promotes locomotor recovery after complete transection of the spinal cord	Journal of Neuroscience	376
36	Kumar et al. (2007)	2007	Molecular basis for the nerve dependence of limb regeneration in an adult vertebrate	Science	374
37	Yamashita and Tohyama (2003)	2003	The p75 receptor acts as a displacement factor that releases Rho from Rho-GDI	Nature Neuroscience	367
38	Raivich et al. (2004)	2004	The AP-1 transcription factor c-jun is required for efficient axonal regeneration	Neuron	364
39	Xin et al. (2017)	2017	MicroRNA cluster miR-17-92 Cluster in Exosomes Enhance Neuroplasticity and Functional Recovery After Stroke in Rats	Stroke	361
40	García-Alías et al. (2009)	2009	Chondroitinase ABC treatment opens a window of opportunity for task-specific rehabilitation	Nature Neuroscience	361
41	Smith et al. (2009)	2009	SOCS3 Deletion Promotes Optic Nerve Regeneration *In Vivo*	NEURON	360
42	MacDonald et al. (2006)	2006	The Drosophila cell corpse engulfment receptor draper mediates glial clearance of severed axons	NEURON	360
43	Tae et al. (2008)	2008	The role of aligned polymer fiber-based constructs in the bridging of long peripheral nerve gaps	Biomaterials	347
44	Dickendesher et al. (2012)	2012	NgR1 and NgR3 are receptors for chondroitin sulfate proteoglycans	Nature Neuroscience	341
45	Oertle et al. (2003)	2003	Nogo-A inhibits neurite outgrowth and cell spreading with three discrete regions	Journal of Neuroscience	337
46	Barritt et al. (2006)	2006	Chondroitinase ABC promotes sprouting of intact and injured spinal systems after spinal cord injury	Journal of Neuroscience	332
47	Willis et al. (2005)	2005	Differential transport and local translation of cytoskeletal, injury-response, and neurodegeneration protein mRNAs in axons	Journal of Neuroscience	331
48	Kim et al. (2003)	2003	Axon regeneration in young adult mice lacking Nogo-A/B	Neuron	328
49	Hammarlund et al. (2009)	2009	Axon Regeneration Requires a Conserved MAP Kinase Pathway	Science	326
50	Park et al. (2005)	2005	A TNF receptor family member, TROY, is a coreceptor with Nogo receptor in mediating the inhibitory activity of myelin inhibitors	Neuron	326
51	Simonen et al. (2003)	2003	Systemic deletion of the myelin-associated outgrowth inhibitor Nogo-A improves regenerative and plastic responses after spinal cord injury	Neuron	326
52	Höke et al. (2006)	2006	Schwann cells express motor and sensory phenotypes that regulate axon regeneration	Journal of Neuroscience	323
53	Shao et al. (2005)	2005	TAJ/TROY, an orphan TNF receptor family member, binds Nogo-66 receptor 1 and regulates axonal regeneration	Neuron	323
54	Lang et al. (2015)	2015	Modulation of the proteoglycan receptor PTPσ promotes recovery after spinal cord injury	Nature	320
55	Corey et al. (2007)	2007	Aligned electrospun nanofibers specify the direction of dorsal root ganglia neurite growth	Journal of Biomedical Materials Research Part A	320
56	Wells (2003)	2003	Neuroprotection by minocycline facilitates significant recovery from spinal cord injury in mice	Brain	320
57	Carmichael et al. (2005)	2005	Growth-associated gene expression after stroke: Evidence for a growth-promoting region in peri-infarct cortex	Experimental Neurology	319
58	Monnier et al. (2003)	2003	The Rho/ROCK pathway mediates neurite growth-inhibitory activity associated with the chondroitin sulfate proteoglycans of the CNS glial scar	Molecular and Cellular Neuroscience	319
59	Duan et al. (2015)	2015	Subtype-Specific Regeneration of Retinal Ganglion Cells following Axotomy: Effects of Osteopontin and mTOR Signaling	Neuron	313
60	Kaneko et al. (2006)	2006	A selective Sema3A inhibitor enhances regenerative responses and functional recovery of the injured spinal cord	Nature Medicine	312
61	Lu et al. (2005)	2005	BDNF-expressing marrow stromal cells support extensive axonal growth at sites of spinal cord injury	Experimental Neurology	311
62	Li et al. (2005)	2005	Gliosis and brain remodeling after treatment of stroke in rats with marrow stromal cells	GLIA	310
63	Verma et al. (2005)	2005	Axonal protein synthesis and degradation are necessary for efficient growth cone regeneration	Journal of Neuroscience	309
64	Lee et al. (2003)	2003	Controlled release of nerve growth factor enhances sciatic nerve regeneration	Experimental Neurology	306
65	Ertürk et al. (2007)	2007	Disorganized microtubules underlie the formation of retraction bulbs and the failure of axonal regeneration	Journal of Neuroscience	304
66	Ruschel et al. (2015)	2015	Systemic administration of epothilone B promotes axon regeneration after spinal cord injury	Science	303
67	Seijffers et al. (2007)	2007	ATF3 increases the intrinsic growth state of DRG neurons to enhance peripheral nerve regeneration	Journal of Neuroscience	300
68	Geremia et al. (2007)	2007	Electrical stimulation promotes sensory neuron regeneration and growth-associated gene expression	Experimental Neurology	300
69	Hara et al. (2017)	2017	Interaction of reactive astrocytes with type I collagen induces astrocytic scar formation through the integrin-N-cadherin pathway after spinal cord injury	Nature Medicine	299
70	Zheng et al. (2003)	2003	Lack of enhanced spinal regeneration in Nogo-deficient mice	Neuron	299
71	Alilain et al. (2011)	2011	Functional regeneration of respiratory pathways after spinal cord injury	Nature	296
72	Lu et al. (2004)	2004	Combinatorial therapy with Neurotrophins and cAMP promotes axonal regeneration beyond sites of spinal cord injury	Journal of Neuroscience	293
73	Koprivica et al. (2005)	2005	EGFR activation mediates inhibition of axon regeneration by myelin and chondroitin sulfate proteoglycans	Science	287
74	Karimi-Abdolrezaee et al. (2010)	2010	Synergistic Effects of Transplanted Adult Neural Stem/Progenitor Cells, Chondroitinase, and Growth Factors Promote Functional Repair and Plasticity of the Chronically Injured Spinal Cord	Journal of neuroscience	283
75	Gao et al. (2004)	2004	Activated CREB is sufficient to overcome inhibitors in myelin and promote spinal axon regeneration *in vivo*	Neuron	282
76	Kim et al. (2004)	2004	Nogo-66 receptor prevents raphespinal and rubrospinal axon regeneration and limits functional recovery from spinal cord injury	Neuron	280
77	Lee et al. (2004)	2004	Nogo receptor antagonism promotes stroke recovery by enhancing axonal plasticity	Journal of Neuroscience	277
78	Prang et al. (2006)	2006	The promotion of oriented axonal regrowth in the injured spinal cord by alginate-based anisotropic capillary hydrogels	Biomaterials	274
79	Nikulina et al. (2004)	2004	The phosphodiesterase inhibitor rolipram delivered after a spinal cord lesion promotes axonal regeneration and functional recovery	Proceedings of the National Academy of Sciences of the United States of America	273
80	Whitlock et al. (2009)	2009	Processed allografts and type I collagen conduits for repair of peripheral nerve gaps	Muscle and Nerve	272
81	Anderson et al. (2018)	2018	Required growth facilitators propel axon regeneration across complete spinal cord injury	Nature	270
82	Lee et al. (2010)	2010	Bio-printing of collagen and VEGF-releasing fibrin gel scaffolds for neural stem cell culture	Experimental Neurology	267
83	Di Summa et al. (2010)	2010	Adipose-derived stem cells enhance peripheral nerve regeneration	Journal of Plastic Reconstructive and Esthetic Surgery	266
84	Li et al. (2004)	2004	Blockade of Nogo-66, myelin-associated glycoprotein, and oligodendrocyte myelin glycoprotein by soluble Nogo-66 receptor promotes axonal sprouting and recovery after spinal injury	Journal of Neuroscience	266
85	Neuhuber et al. (2005)	2005	Axon growth and recovery of function supported by human bone marrow stromal cells in the injured spinal cord exhibit donor variations	BRAIN RESEARCH	265
86	Lu et al. (2014)	2014	Long-Distance Axonal Growth from Human Induced Pluripotent Stem Cells after Spinal Cord Injury	Neuron	263
87	Lopez-Verrilli et al. (2013)	2013	Schwann Cell-Derived Exosomes Enhance Axonal Regeneration in the Peripheral Nervous System	GLIA	263
88	Yan et al. (2009)	2009	The DLK-1 Kinase Promotes mRNA Stability and Local Translation in *C. elegans* Synapses and Axon Regeneration	CELL	263
89	Liebscher et al. (2005)	2005	Nogo-A antibody improves regeneration and locomotion of spinal cord-injured rats	Annals of Neurology	262
90	Ertürk et al. (2012)	2012	Three-dimensional imaging of the unsectioned adult spinal cord to assess axon regeneration and glial responses after injury	Nature Medicine	261
91	Goldshmit et al. (2004)	2004	Axonal regeneration and lack of astrocytic gliosis in EphA4-deficient mice	Journal of Neuroscience	260
92	Menet et al. (2003)	2003	Axonal plasticity and functional recovery after spinal cord injury in mice deficient in both glial fibrillary acidic protein and vimentin genes	Proceedings of the National Academy of Sciences of the United States of America	260
93	Mead and Tomarev (2017)	2017	Bone Marrow-Derived Mesenchymal Stem Cells-Derived Exosomes Promote Survival of Retinal Ganglion Cells Through miRNA-Dependent Mechanisms	Stem Cells Translational Medicine	258
94	Christie et al. (2010)	2010	PTEN Inhibition to Facilitate Intrinsic Regenerative Outgrowth of Adult Peripheral Axons	Journal of Neuroscience	258
95	Rivieccio et al. (2009)	2009	HDAC6 is a target for protection and regeneration following injury in the nervous system	Proceedings of the National Academy of Sciences of the United States of America	255
96	Sivasankaran et al. (2004)	2004	PKC mediates inhibitory effects of myelin and chondroitin sulfate proteoglycans on axonal regeneration	Nature Neuroscience	253
97	Li and Strittmatter (2003)	2003	Delayed systemic Nogo-66 receptor antagonist promotes recovery from spinal cord injury	Journal of Neuroscience	247
98	Chen et al. (2007)	2007	Transplantation of bone marrow stromal cells for peripheral nerve repair	Experimental Neurology	245
99	Hudson et al. (2004)	2004	Engineering an improved acellular nerve graft via optimized chemical processing	Tissue Engineering	235
100	Ankeny et al. (2004)	2004	Bone marrow transplants provide tissue protection and directional guidance for axons after contusive spinal cord injury in rats	Experimental Neurology	215

### 3.2 Analysis of the most productive countries

**Figure 3:**
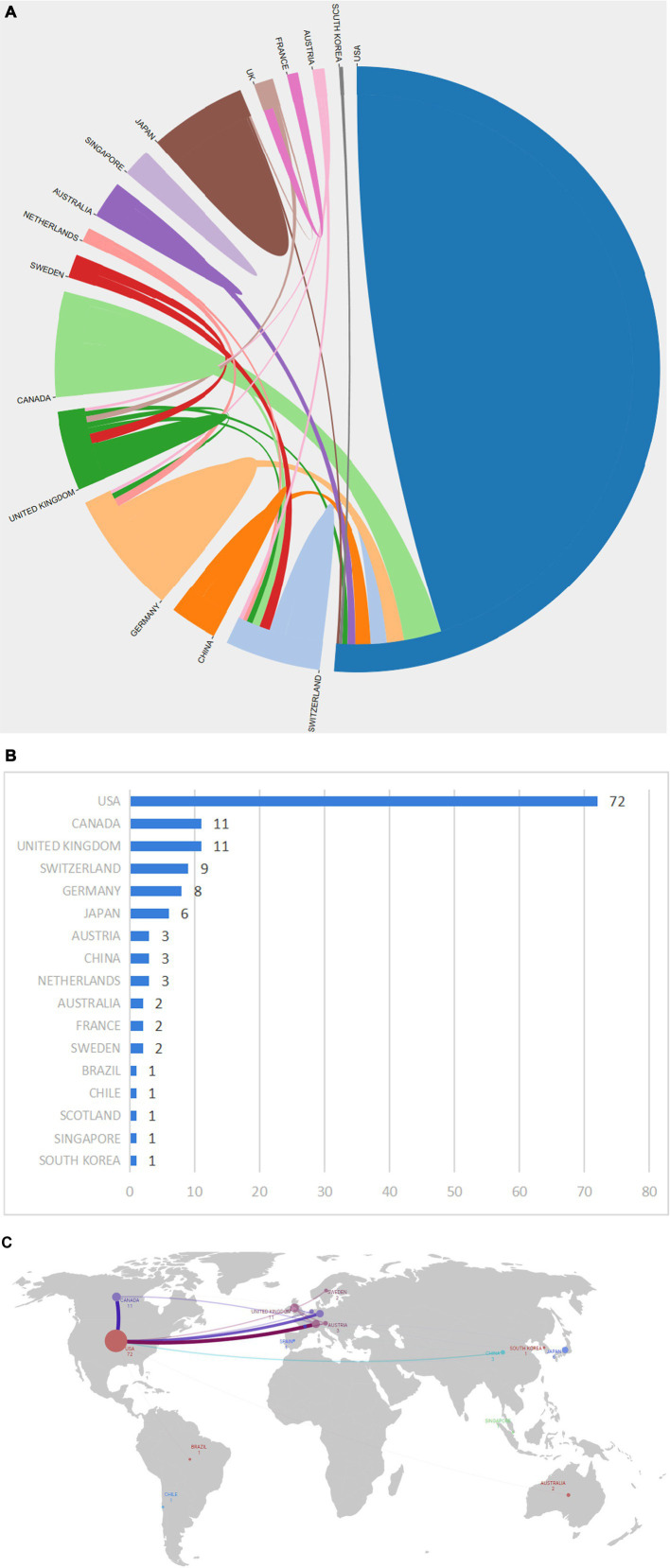
The most productive countries. **(A)** A network map of the countries involved in this research area. **(B)** The number of publications by country. **(C)** The world map displays the number of national publications, the larger the dots, the greater the number of articles published by a country. The lines in **(C)** denote the presence of cooperative relationships between countries.

**Figure 4:**
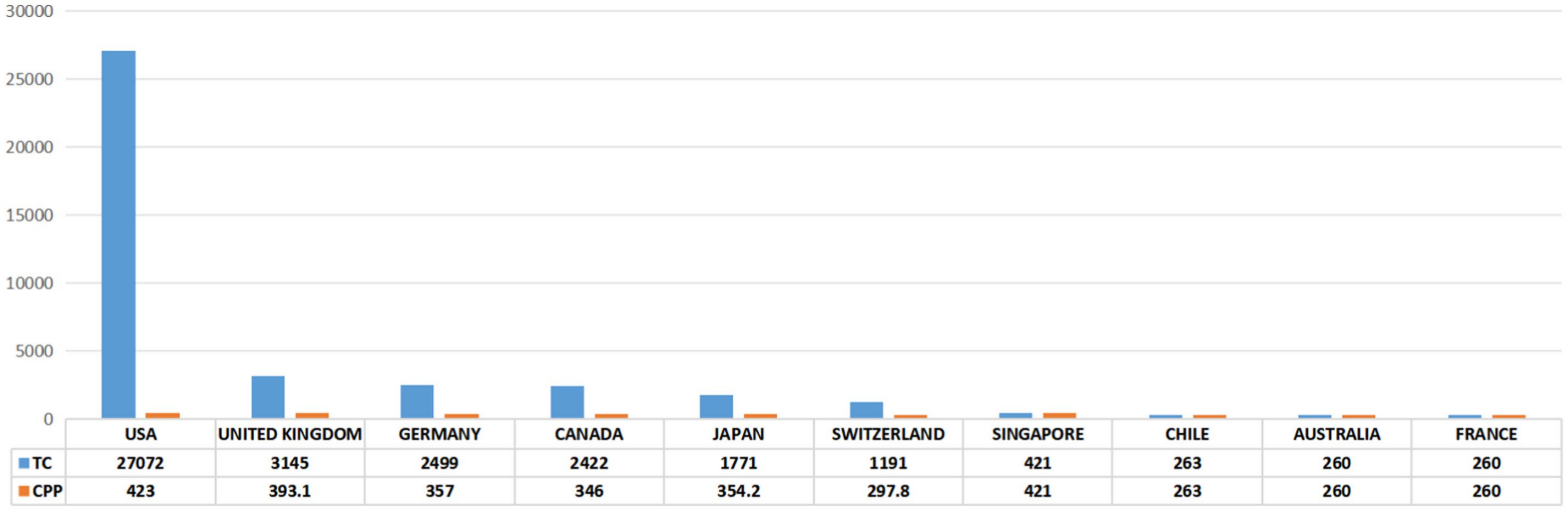
The 10 countries with the most publications. TC, total citations. CPP, citations per publication.

As shown in Figures 3A,B, a total of 16 countries/regions contributed to the 100 most cited papers, with 6 of these countries/regions contributing more than 3 articles. Scimago Graphica software was employed to generate a world map highlighting the countries associated with the top 100 cited publications (Figure 3C). The field of axon regeneration was dominated by the USA, with 72 out of the 100 most cited papers, 27,072 citations, and an average citation count of 423, which was the highest among all the countries (Figures 3, 4). The United Kingdom was ranked second, contributing 11 papers and receiving 3,145 citations, with an average citation count of 393.1. Germany was the third-ranked country, contributing 8 articles and receiving 2,499 citations, with an average citation count of 357.

### 3.3 Institution analysis

**Figure 5:**
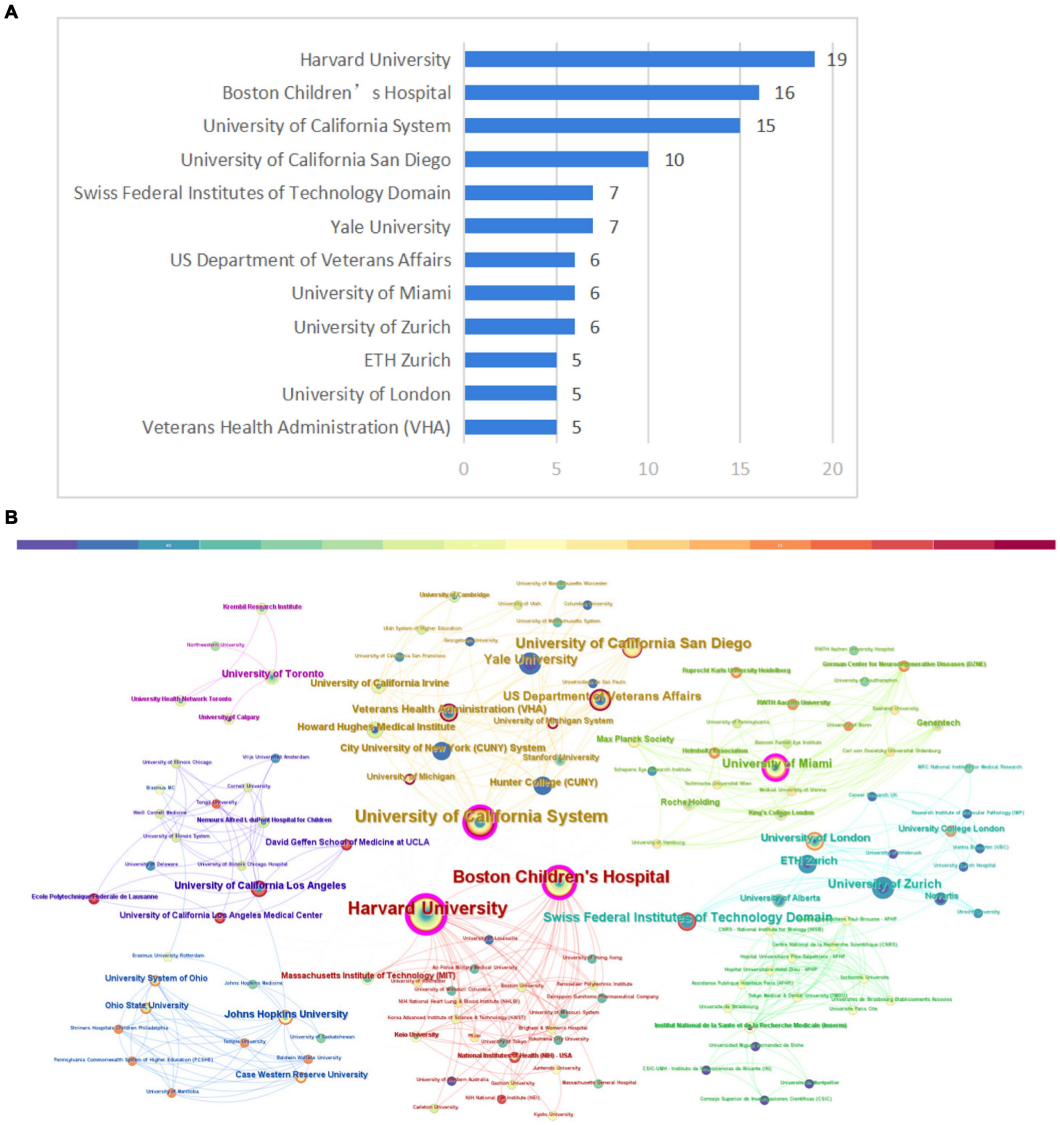
Institution analysis. **(A)** The most relevant institutions. **(B)** Partnerships between institutions. The size of the circle in the graph corresponds to the number of relevant publications originating from the institution and the lines signify collaborative relationships between institutions.

In total, 155 institutions contributed to the top 100 cited articles relating to axon regeneration. Figure 5A shows the publication count for the most pertinent organizations. Among these, Harvard University emerged as the leading institution, generating the highest number of top-cited articles (*n* = 19). CiteSpace software was used to visualize the interconnections between institutions (Figure 5B). Several prominent research institutions, such as Harvard University, Boston Children’s Hospital, the University of California, and the University of Miami, among others, can be identified.

### 3.4 Author analysis

**Figure 6:**
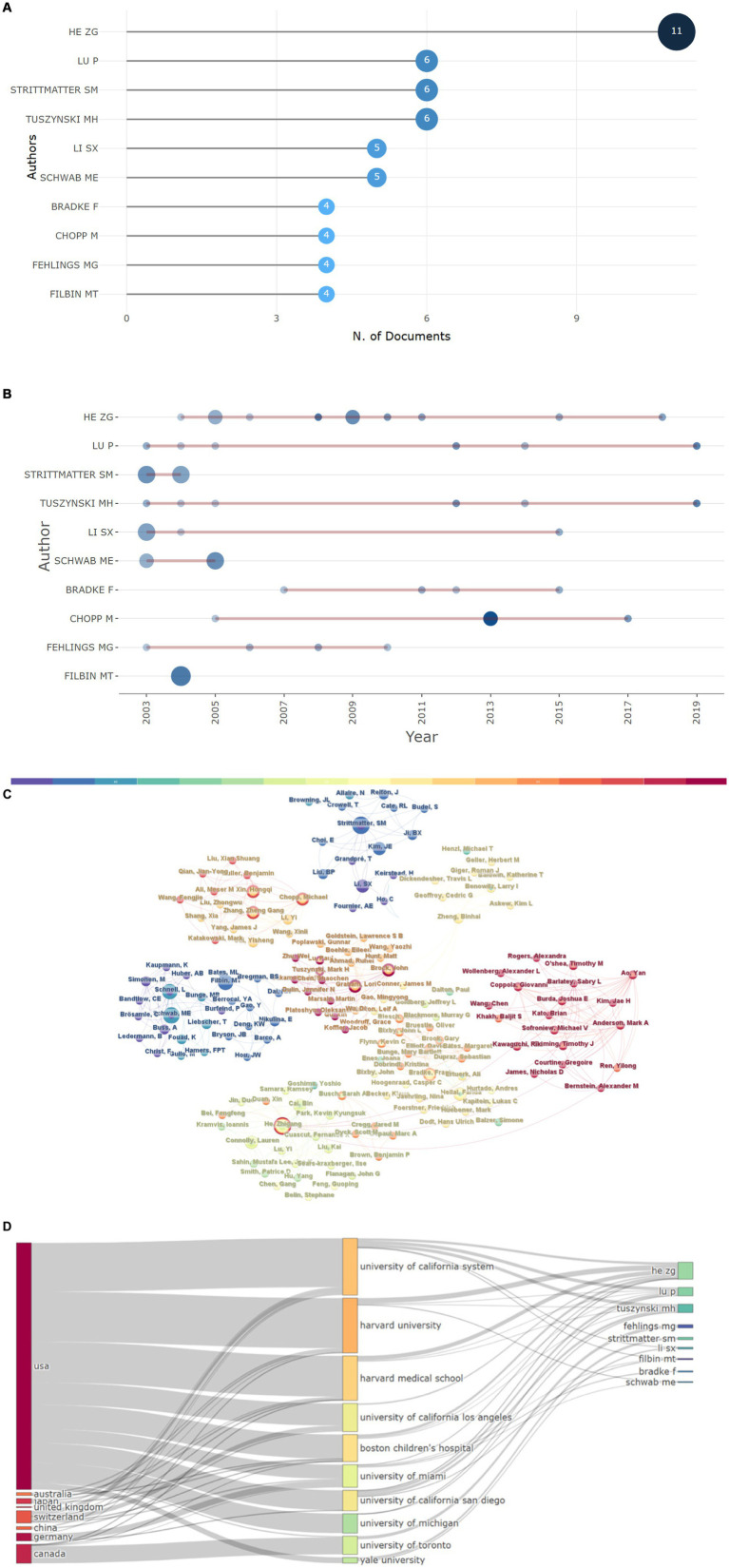
Author analysis. **(A)** The 10 most relevant authors and **(B)** their publications over time. **(C)** Author collaborative relationship map, the size of each node correlates with the number of articles among the top 100 articles in this field published by an author; the lines denote collaborative relationships among authors. **(D)** Three-field plot of the relationships among countries, institutions, and authors.

The most relevant authors were identified by employing the “bibliometrix” R package. As demonstrated in Figure 6A, He, Zhigang had the highest number of publications (*n* = 11). Figure 6B depicts the timeline of publications for the 10 most influential authors. Additionally, our analysis indicated that author collaboration was relatively frequent, and partnerships between authors were consistent (Figure 6C). The relationships among countries, institutions, and authors are shown in Figure 6D, with authors from the USA having a substantial impact in this respect.

### 3.5 Analysis of journals

Table 2 displays the journals in which the 100 most cited articles were published. The Journal of Neuroscience published the highest number of articles (*n* = 21), with 8,510 citations. This was followed by Neuron, with 12 publications and 3,824 citations; Experimental Neurology, which published 10 of the 100 papers and had 3,623 citations; and Science, which contributed 9 articles and received 4,345 citations, with an average citation count of 482.78. Furthermore, 9 journals published more than 3 articles—Nature Neuroscience, Nature Medicine, Biomaterials, Nature, and Proceedings of the National Academy of Sciences of the United States of America, which accumulated 3,465, 2,252, 2,201, 2,546, and 1,825 citations, respectively.

**Table 2:** Journals of the 100 most-cited articles in the field of axon regeneration.

Ranking	Journal	Documents	Total citations	IF in 2022	5 Year IF	Average citations
1	Journal of Neuroscience	21	8,510	5.3	6.2	405.24
2	Neuron	12	3,824	16.2	18.6	318.67
3	Experimental Neurology	10	3,623	5.3	5.1	362.30
4	Science	9	4,345	56.9	54.5	482.78
5	Nature Neuroscience	8	3,465	25	27.7	433.13
6	Nature Medicine	6	2,252	82.9	69.4	375.33
7	Biomaterials	5	2,201	14	13.8	440.20
7	Nature	5	2,546	64.8	60.9	509.20
7	Proceedings of the National Academy of Sciences of the United States of America	5	1825	11.1	12	365.00
10	Cell	3	1,440	64.5	57.5	480.00
11	Brain Research	2	745	2.9	3.2	372.50
11	GLIA	2	573	6.2	7	286.50
13	Annals of Neurology	1	262	11.2	11.4	262.00
13	BRAIN	1	320	14.5	14.6	320.00
13	Journal of Biomedical Materials Research Part A	1	320	4.9	4.6	320.00
13	Journal of Cerebral Blood Flow and Metabolism	1	674	6.3	6.3	674.00
13	Journal of Clinical Investigation	1	399	15.9	16.7	399.00
13	Journal of Plastic Reconstructive and Esthetic Surgery	1	266	2.7	2.9	266.00

### 3.6 Keywords and research hotspots

**Figure 7:**
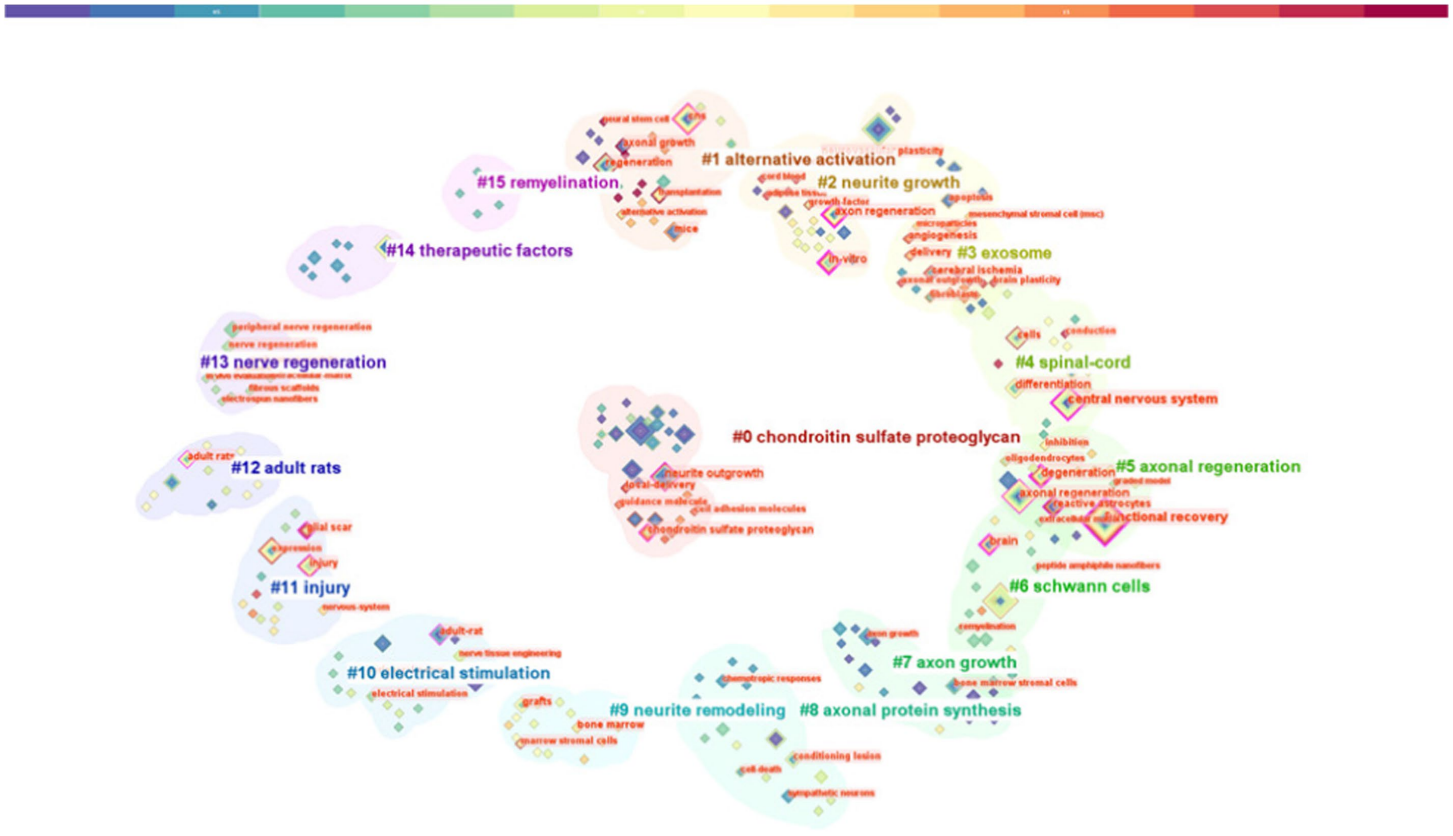
Keyword analysis: keyword cluster graph.

**Figure 8:**
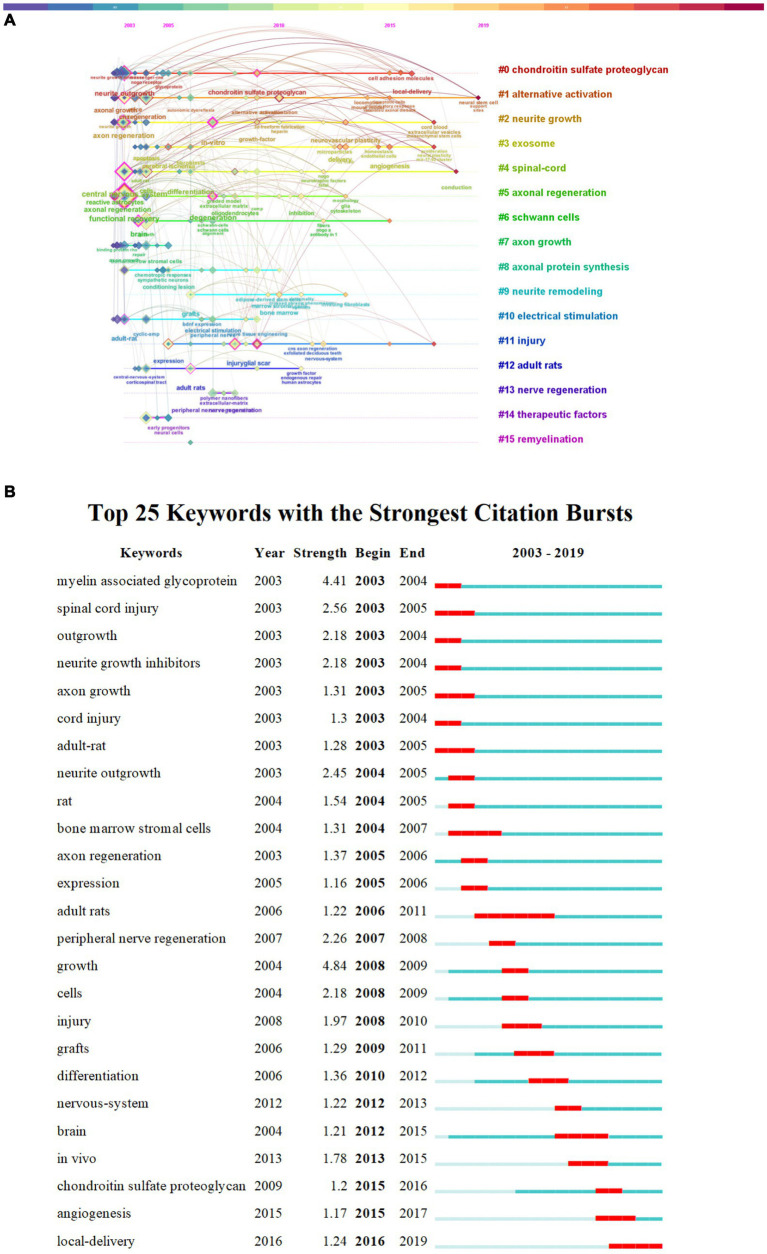
Keyword changes over time. **(A)** Keyword timeline. **(B)** Keyword burst analysis.

Keywords play a crucial role in delineating the focus of an article, thereby providing researchers with a comprehensive understanding of the published subject matter. The co-occurrence of two keywords within a given paper signifies that there is an inherent relationship between them, with the frequency of their appearance denoting the intensity of this association. By undertaking keyword co-occurrence and emergent item analysis, we can discern hot topics within a specific field across varying periods, and aggregate the keywords provided by authors in the dataset. Keyword clustering characterizes the inherent knowledge structure within a specific research field and categorizes its domain. In this study, cluster analysis revealed that keywords within the field of axon regeneration could be partitioned into 16 categories (Figure 7) —“chondroitin sulfate proteoglycan,” “alternative activation,” “neurite growth,” “exosome,” “spinal cord,” “axonal regeneration,” “Schwann cells,” “axon growth,” “axonal protein synthesis,” “neurite remodeling,” “electrical stimulation,” “injury,” “adult rats,” “nerve regeneration,” “therapeutic factors,” and “remyelination.” Figure 8 illustrates the trend in keyword changes over time. The dynamic relationship between keywords and time is portrayed in Figure 8A. The size of each block relates to the popularity of the respective keyword, the larger the block the higher the frequency of keyword occurrence. Moreover, keywords exhibiting a recent increasing trend may represent popular future research topics.

We conducted a burst analysis of the keywords associated with axon regeneration and identified several prominent keywords including growth myelin-associated glycoprotein spinal cord injury neurite outgrowth peripheral nerve regeneration outgrowth neurite growth inhibitors and cells. Keywords with the highest burst strength may represent the forefront of research in a given field. Keywords with earlier bursts are likely to have been the focus of research in the early years whereas those with more recent bursts represent topics exhibiting a sudden surge in attention

As depicted in Figure 8B, the five keywords with the greatest burst strengths were “growth,” “myelin-associated glycoprotein,” “spinal cord injury,” “neurite outgrowth,” and “peripheral nerve regeneration,” showing burst strengths of 4.84, 4.41, 2.56, 2.45, and 2.26, respectively. The earliest bursting keywords were “myelin-associated glycoprotein,” “spinal cord injury,” “outgrowth,” “neurite growth inhibitors,” “axon growth,” and “adult rat,” while the most recent bursting keywords comprised “local delivery.”

## 4 Discussion

In this study, we conducted a bibliometric analysis of the 100 most influential publications in axon regeneration. This methodological approach allowed us to examine the dynamics, focal points, and cutting-edge aspects of axon regeneration, and provided crucial quantitative information regarding both classical studies and recent advances relating to this process, thereby enhancing our understanding in this field. We also analyze the trends and hotspots of the axon regeneration research, and we believe that these trends and hotspots will point out the direction for future research on SCI treatment.

In total, 39,548 citations were amassed by the analyzed pool of 100 papers, with individual citation counts for the top 100 articles fluctuating between 215 and 1,604, with a median of 326. He, Zhigang emerged as the most prolific author, having authored 8 papers within this collection. Most of the publications originated from the United States, with a count of 72, followed by Canada and the United Kingdom with 11 papers each. Harvard University was the institution with the highest contribution, generating 19 papers, followed by Boston Children’s Hospital with 16 papers and the University of California with 15. Keyword analysis revealed several research areas of interest, including chondroitin sulfate proteoglycan, alternative activation, exosome, Schwann cells, axonal protein synthesis, electrical stimulation, therapeutic factors, and remyelination. The examination of keywords in the articles further indicated that the most recent prominent keyword was “local delivery.”

### 4.1 Chondroitin sulfate proteoglycan

Chondroitin sulfate proteoglycans (CSPGs) comprise a class of large molecules ubiquitously present in the CNS that play pivotal roles in neural development, synaptic plasticity, and neural injury repair. However, CSPGs tend to accumulate in substantial quantities at neural injury sites, thereby establishing an inhibitory milieu that impedes axon regeneration (Silver and Miller, 2004), and surmounting this inhibitory activity of CSPGs to achieve axon regeneration represents a significant challenge. CSPGs hinder axonal growth through their interaction with a range of receptors, including Nogo receptor (NgR) and protein tyrosine phosphatase sigma (PTPσ). Following their activation on neurons, these receptors trigger a cascade of signal transduction pathways, culminating in cytoskeletal reorganization and the consequent inhibition of axonal growth (Shen et al., 2009). Nevertheless, recent research has uncovered multiple strategies to counteract the inhibitory impact of CSPGs and foster axon regeneration. These include employing the enzyme chondroitinase ABC (ChABC) to degrade CSPGs, which nullifies their inhibitory effect on axonal growth (Bradbury et al., 2002), and obstructing the interactions between CSPGs and their receptors or intervening in the signal transduction pathways activated by CSPGs (Monnier et al., 2003). In summary, the accumulation of CSPGs post-neural injury engenders an environment that curbs axon regeneration. Nevertheless, this inhibitory effect of CSPGs can be surmounted, thereby facilitating axon regeneration, through their degradation, by obstructing their interaction with receptors, or by intervening in their signaling pathways. These findings provide novel therapeutic strategies for the repair of neurons after injury.

### 4.2 Alternative activation

The term “alternative activation” generally refers to the activation state of immune cells, specifically macrophages. Following neural injury, the behavior of macrophages and other immune cells substantially impacts the potential for axon regeneration. Macrophages are multifunctional immune cells that can adopt diverse activation states contingent on environmental cues. These activation states comprise two primary categories, namely, M1 and M2. M1 macrophages primarily contribute to inflammation and immune responses, whereas M2 (also termed “alternatively activated”) macrophages participate in repair and regenerative processes (Kigerl et al., 2009). Following neural injury, M1 macrophages aggregate at the injury site and secrete abundant inflammatory mediators and cytokines, which can exacerbate neuronal damage and hinder axon regeneration. Conversely, factors synthesized by M2 macrophages can foster neuronal survival and axonal expansion, thereby facilitating neuronal repair. These observations suggest that axon regeneration can be promoted by modulating the activation state of macrophages. For instance, inducing the transition of macrophages toward the M2 phenotype might assist in enhancing repair following neural injury (Gensel and Zhang, 2015). Although the viability of this strategy has been corroborated in some experimental models, its implementation in clinical settings requires further investigation.

### 4.3 Schwann cells

Schwann cells are the primary glial cells in the PNS and play a crucial role in axon regeneration. Their key functions in this process include the following: (1) The provision of growth factors. Schwann cells can produce and release a variety of growth factors, such as nerve growth factor (NGF), brain-derived neurotrophic factor (BDNF), and neurotrophin-3 (NT-3), which can promote axonal growth and regeneration (Kingham et al., 2007). (2) The formation of regeneration tracks: Following peripheral nerve injury, Schwann cells can proliferate and align into bundles, forming so-called “regeneration tracks” or “Bands of Büngner” that provide physical support and guidance cues for regenerating axons (Fouad et al., 2005). (3) Myelination: Schwann cells can form myelin, a lipid-rich membrane that wraps around axons, providing electrical insulation and accelerating nerve impulse conduction. (4) Immune regulation: Schwann cells can modulate immune responses by secreting a variety of cytokines that can influence the inflammatory response and neuron repair following nerve injury. These functions of Schwann cells underscore their pivotal role in the repair process following nervous system insult.

### 4.4 Exosome

Exosomes are diminutive vesicles produced by living cells that are subsequently released into the extracellular environment. They can carry molecules, including proteins, lipids, and RNAs, which are transferred to recipient cells following their internalization, a process that represents a form of intercellular communication. Evidence increasingly suggests that exosomes play a pivotal role in neural repair and axon regeneration through the delivery of growth-promoting molecules. For example, it has been shown that exosomes produced by Schwann cells can carry and deliver growth factors, enzymes, and other molecules that can promote axon regeneration and provide neuroprotection (Lopez-Verrilli et al., 2013). Although these preliminary research findings hold promise for achieving axon repair, our understanding of the specific mechanisms by which exosomes influence axon regeneration remains inadequate. Further comprehensive research is required to address this limitation and determine the potential of exosomes in neural repair.

### 4.5 Axonal protein synthesis

Protein synthesis within axons is vital for axonal growth and regeneration. It was originally thought that most protein synthesis occurred in the cell body, after which the synthesized proteins would be transported to the axon via axoplasmic transport; however, it is now acknowledged that there exists a mechanism for local protein synthesis within the axon itself, which allows neurons to rapidly respond to environmental stimuli. The possible roles of this mechanism include the following: (1) Supporting axonal growth. Within the growth cone of axons, local protein synthesis can quickly provide the proteins required for axonal growth. For example, the local translation of β-actin mRNA was found to be crucial for axonal growth and guidance (Jung et al., 2012). (2) Responding to environmental signals. Axons can rapidly respond to environmental signals through local protein synthesis. For example, some growth factors and guidance molecules can stimulate the translation of specific mRNAs in axons, thereby affecting axonal growth and guidance. (3) Regeneration following nerve injury. Following nerve injury, local protein synthesis may be crucial for axonal regeneration. For example, local protein synthesis in axons increases after nerve injury, which may facilitate axonal regeneration (Twiss and Fainzilber, 2009).

Despite our limited understanding of local protein synthesis in axons, research within this domain presents novel possibilities for developing strategies for neural regeneration therapy.

### 4.6 Electrical stimulation

Electrical stimulation has been demonstrated to foster axon regeneration and has been utilized in the treatment of certain nerve injuries. The potential underlying mechanisms include the following: (1) Increasing nerve growth factor expression. Electrical stimulation can enhance the expression of nerve growth factors (such as NGF and BDNF, among others), which can promote axonal growth and regeneration. (2) Modifying the electrophysiological activity of nerve cells. Electrical stimulation can alter the electrophysiological activity of nerve cells by, for example, modifying the activity of ion channels and increasing the excitability of nerve cells, thereby promoting axonal growth and regeneration. (3) Manipulating intracellular signaling pathways. Electrical stimulation can influence intracellular signaling pathways, such as increasing the level of cAMP and activating the mTOR signaling pathway, both of which play a key role in axonal growth and regeneration. (4) Enhancing angiogenesis and ameliorating the local microenvironment. Electrical stimulation can also promote angiogenesis and improve the microenvironment of the nerve injury area, providing favorable conditions for axonal growth and regeneration.

Although the facilitating effect of electrical stimulation on axon regeneration has been widely confirmed, further research is needed to elucidate in detail the specific mechanisms of action. Furthermore, the need to optimize electrical stimulation parameters, such as the intensity, frequency, and duration of stimulation, and thereby achieve the most beneficial therapeutic effect, is also a significant issue in current research.

### 4.7 Remyelination

Remyelination and axon regeneration constitute two closely related pivotal steps in the process of neural repair. An axon is a protrusion of a neuron through which information is transmitted to adjacent cells. The axons of many types of neurons are enveloped in a lipid structure called myelin, which serves both to accelerate the propagation of nerve impulses and protect the axon. In the CNS, myelin is produced by oligodendrocytes, while in the PNS, it is produced by Schwann cells. Following nerve injury, axons might be severed, and myelin may be lost or damaged. Despite their importance for nerve repair, axon regeneration and remyelination do not invariably proceed in synchrony. For example, axons may grow without sufficient myelin, or myelin may form without axons. Remyelination and axon regeneration represent intertwined processes, with each influencing the other. For instance, during axon regeneration, neurons release factors, such as NGF, that stimulate the proliferation and differentiation of Schwann cells or oligodendrocytes, thereby promoting remyelination (Höke et al., 2006). Regarding the effect of remyelination on axon regeneration, in addition to forming myelin, myelinating cells can also secrete factors, such as neurotrophic factors, that promote axon growth. Moreover, myelin-forming cells can also provide a structural framework for guiding axon growth through physical contact with growing axons (Höke et al., 2006; Kingham et al., 2007). In the treatment of nerve injury, strategies to promote both remyelination and axon regeneration are often considered simultaneously.

### 4.8 Local delivery

The development of local delivery systems has emerged as a particularly promising strategy for promoting axon regeneration, allowing for the sustained and targeted release of therapeutic agents directly at the site of injury. These systems, including hydrogels, nanoparticles, and biomaterials, have been engineered to provide a supportive environment for axonal growth as well as to overcome the inhibitory effects of the glial scar. Lu et al. (2003) and Xin et al. (2013) demonstrated the neurotrophic factor-secreting potential of neural stem cells and mesenchymal stem cells, and that these cells can be leveraged for local delivery, fostering a conducive environment for axonal regeneration. After nerve injury, especially after SCI, the local microenvironment, such as inflammation and scar tissue formation at the injury site, is one of the important factors that hinder its repair. If the local delivery of chemical drugs or bioactive molecules can effectively improve the inhibitory local microenvironment, it will be very beneficial to the treatment of SCI. Thus, with the increasing development of “local delivery” technique, this technique will be widely used in SCI repair, by reducing the dose of drugs, to achieve better efficacy, while reducing its toxic side effects.

### 4.9 Clinical translation

A collaborative, multidisciplinary approach that integrates molecular biology, bioengineering, and clinical neuroscience will be crucial for developing effective treatments. Standardized protocols for preclinical studies and the establishment of sensitive biomarkers will facilitate the translation of research findings into clinical practice, ultimately improving patient outcomes following CNS insult. The continued exploration of the intricate balance between regeneration-promoting and regeneration-inhibiting factors, along with the optimization of local delivery systems, will be instrumental in realizing the full potential of axon regeneration therapies. However, although our animal experiments have yielded encouraging results, compare to human, animals are more compensatory after SCI, and walking on four legs can easily lead to misjudgments in behavioral assessments. Therefore, more primate experiments should be conducted in the future, and more sophisticated behavioral assessment methods should be developed.

### 4.10 Study limitations

This study had several limitations. Despite being the most used database for literature searches, Web of Science does not encompass all publications. To ensure the accuracy of our analysis, we employed the topic search method instead of the subject keywords search method. Although our search results demonstrate some precision, they could have been more comprehensive.

## 5 Conclusion

The extensive body of research on axon regeneration, as reflected in the 100 most cited articles, has highlighted a multifaceted approach for promoting the repair and regeneration of damaged axons in the CNS. This research has underscored the importance of intrinsic neuronal growth capabilities, the modulation of the PTEN/mTOR pathway, the role of astrocyte scar formation, and the potential of stem cell therapies in neuronal repair after injury. The identification of distinct macrophage subsets with divergent effects on neurotoxicity or regeneration, as demonstrated by Kigerl et al. (2009), has added a layer of complexity to our understanding of the response to injury and the potential for targeted interventions. Despite the progress to date, several challenges concerning the ability to promote axon regeneration persist. Significant hurdles include the heterogeneity of injury models, the lack of standardized outcome measures, and the lack of robust biomarkers to assess the success of regenerative therapies. The translation of preclinical research findings into a clinical setting is further complicated by the complexity of the CNS environment.

This study offers bibliometric insights into axon regeneration, underscoring that the United States is a prominent leader in this field. Our analysis highlights the growing relevance of local delivery systems in axon regeneration. Although these systems have shown promise in preclinical models, challenges in long-term optimization, agent selection, and clinical translation remain. Despite these hurdles, the continued development of local delivery technologies represents a promising pathway for achieving axon regeneration; however, further research is essential to fully realize their potential and enhance patient outcomes.

## Author contributions

Saijilafu: Writing – original draft, Writing – review & editing. L-CY: Writing – original draft. J-YZ: Investigation, Methodology, Writing – review & editing. R-JX: Conceptualization, Supervision, Writing – review & editing.

## Footnotes

^1^https://bibliometric.com/

## References

[ref1] Alilain W. J. Horn K. P. Hu H. Dick T. E. Silver J. (2011). Functional regeneration of respiratory pathways after spinal cord injury. Nature 475, 196–200. doi: 10.1038/nature10199, PMID: 21753849 PMC3163458

[ref2] Anderson M. Burda J. Ren Y. Ao Y. O’Shea T. M. Kawaguchi R.. (2016). Astrocyte scar formation aids central nervous system axon regeneration. Nature 532, 195–200. doi: 10.1038/nature17623, PMID: 27027288 PMC5243141

[ref3] Anderson M. A. O’Shea T. M. Burda J. E. Ao Y. Barlatey S. L. Bernstein A. M.. (2018). Required growth facilitators propel axon regeneration across complete spinal cord injury. Nature 561, 396–400. doi: 10.1038/s41586-018-0467-6, PMID: 30158698 PMC6151128

[ref4] Ankeny D. P. McTigue D. M. Jakeman L. B. (2004). Bone marrow transplants provide tissue protection and directional guidance for axons after contusive spinal cord injury in rats. Exp. Neurol. 190, 17–31. doi: 10.1016/j.expneurol.2004.05.045, PMID: 15473977

[ref5] Atwal J. K. Pinkston-Gosse J. Syken J. Stawicki S. Wu Y. Shatz C.. (2008). PirB is a functional receptor for myelin inhibitors of axonal regeneration. Science 322, 967–970. doi: 10.1126/science.1161151 18988857

[ref6] Barnabé-Heider F. Frisén J. (2008). Stem cells for spinal cord repair. Cell Stem Cell 3, 16–24. doi: 10.1016/j.stem.2008.06.011 18593555

[ref7] Barritt A. W. Davies M. Marchand F. Hartley R. Grist J. Yip P.. (2006). Chondroitinase ABC promotes sprouting of intact and injured spinal systems after spinal cord injury. J. Neurosci. 26, 10856–10867. doi: 10.1523/JNEUROSCI.2980-06.2006, PMID: 17050723 PMC3339436

[ref8] Bradbury E. Moon L. Popat R. King V. R. Bennett G. S. Patel P. N.. (2002). Chondroitinase ABC promotes functional recovery after spinal cord injury. Nature 416, 636–640. doi: 10.1038/416636a 11948352

[ref9] Carmichael S. T. Archibeque I. Luke L. Nolan T. Momiy J. Li S. (2005). Growth-associated gene expression after stroke: evidence for a growth-promoting region in peri-infarct cortex. Exp. Neurol. 193, 291–311. doi: 10.1016/j.expneurol.2005.01.004 15869933

[ref10] Cattin A. L. Burden J. J. van Emmenis L. Mackenzie F. E. Hoving J. J. A. Garcia Calavia N.. (2015). Macrophage-induced Blood vessels guide Schwann cell-mediated regeneration of peripheral nerves. Cell 162, 1127–1139. doi: 10.1016/j.cell.2015.07.021, PMID: 26279190 PMC4553238

[ref11] Chen C. (2004). Searching for intellectual turning points: progressive knowledge domain visualization. Proc. Natl. Acad. Sci. USA 101, 5303–5310. doi: 10.1073/pnas.0307513100 14724295 PMC387312

[ref12] Chen C. J. Ou Y. C. Liao S. L. Chen W. Y. Chen S. Y. Wu C. W.. (2007). Transplantation of bone marrow stromal cells for peripheral nerve repair. Exp. Neurol. 204, 443–453. doi: 10.1016/j.expneurol.2006.12.004 17222827

[ref13] Christie K. J. Webber C. A. Martinez J. A. Singh B. Zochodne D. W. (2010). PTEN inhibition to facilitate intrinsic regenerative outgrowth of adult peripheral axons. J. Neurosci. 30, 9306–9315. doi: 10.1523/JNEUROSCI.6271-09.2010, PMID: 20610765 PMC6632469

[ref14] Corey J. M. Lin D. Y. Mycek K. B. Chen Q. Samuel S. Feldman E. L.. (2007). Aligned electrospun nanofibers specify the direction of dorsal root ganglia neurite growth. J. Biomed. Mater. Res. 83A, 636–645. doi: 10.1002/jbm.a.31285, PMID: 17508416

[ref15] Crigler L. Robey R. C. Asawachaicharn A. Gaupp D. Phinney D. G. (2006). Human mesenchymal stem cell subpopulations express a variety of neuro-regulatory molecules and promote neuronal cell survival and neuritogenesis. Exp. Neurol. 198, 54–64. doi: 10.1016/j.expneurol.2005.10.029, PMID: 16336965

[ref16] Di Summa P. G. Kingham P. J. Raffoul W. Wiberg M. Terenghi G. Kalbermatten D. F. (2010). Adipose-derived stem cells enhance peripheral nerve regeneration. J. Plast. Reconstr. Aesthet. Surg. 63, 1544–1552. doi: 10.1016/j.bjps.2009.09.012 19828391

[ref17] Dickendesher T. L. Baldwin K. T. Mironova Y. A. Koriyama Y. Raiker S. J. Askew K. L.. (2012). NgR1 and NgR3 are receptors for chondroitin sulfate proteoglycans. Nat. Neurosci. 15, 703–712. doi: 10.1038/nn.3070, PMID: 22406547 PMC3337880

[ref18] Duan X. Qiao M. Bei F. Kim I. J. He Z. Sanes J. R. (2015). Subtype-specific regeneration of retinal ganglion cells following Axotomy: effects of Osteopontin and mTOR signaling. Neuron 85, 1244–1256. doi: 10.1016/j.neuron.2015.02.017, PMID: 25754821 PMC4391013

[ref19] Ellis-Behnke R. G. Liang Y. X. You S. W. Tay D. K. C. Zhang S. So K. F.. (2006). Nano neuro knitting: peptide nanofiber scaffold for brain repair and axon regeneration with functional return of vision. Proc. Natl. Acad. Sci. USA 103, 5054–5059. doi: 10.1073/pnas.0600559103, PMID: 16549776 PMC1405623

[ref20] Ertürk A. Hellal F. Enes J. Bradke F. (2007). Disorganized microtubules underlie the formation of retraction bulbs and the failure of axonal regeneration. J. Neurosci. 27, 9169–9180. doi: 10.1523/JNEUROSCI.0612-07.2007, PMID: 17715353 PMC6672197

[ref21] Ertürk A. Mauch C. P. Hellal F. Förstner F. Keck T. Becker K.. (2012). Three-dimensional imaging of the unsectioned adult spinal cord to assess axon regeneration and glial responses after injury. Nat. Med. 18, 166–171. doi: 10.1038/nm.2600, PMID: 22198277

[ref22] Fouad K. Schnell L. Bunge M. B. Schwab M. E. Liebscher T. Pearse D. D. (2005). Combining Schwann cell bridges and olfactory-Ensheathing glia grafts with Chondroitinase promotes locomotor recovery after complete transection of the spinal cord. J. Neurosci. 25, 1169–1178. doi: 10.1523/JNEUROSCI.3562-04.2005, PMID: 15689553 PMC6725952

[ref23] Fournier A. E. Takizawa B. T. Strittmatter S. M. (2003). Rho kinase inhibition enhances axonal regeneration in the injured CNS. J. Neurosci. 23, 1416–1423. doi: 10.1523/JNEUROSCI.23-04-01416.2003, PMID: 12598630 PMC6742251

[ref24] Gao Y. Deng K. Hou J. Bryson J. B. Barco A. Nikulina E.. (2004). Activated CREB is sufficient to overcome inhibitors in myelin and promote spinal axon regeneration in vivo. Neuron 44, 609–621. doi: 10.1016/j.neuron.2004.10.030, PMID: 15541310

[ref25] García-Alías G. Barkhuysen S. Buckle M. Fawcett J. W. (2009). Chondroitinase ABC treatment opens a window of opportunity for task-specific rehabilitation. Nat. Neurosci. 12, 1145–1151. doi: 10.1038/nn.2377 19668200

[ref26] Gensel J. C. Zhang B. (2015). Macrophage activation and its role in repair and pathology after spinal cord injury. Brain Res. 1619, 1–11. doi: 10.1016/j.brainres.2014.12.045, PMID: 25578260

[ref27] Geremia N. M. Gordon T. Brushart T. M. Al-Majed A. A. Verge V. M. K. (2007). Electrical stimulation promotes sensory neuron regeneration and growth-associated gene expression. Exp. Neurol. 205, 347–359. doi: 10.1016/j.expneurol.2007.01.040, PMID: 17428474

[ref28] Goldshmit Y. Galea M. P. Wise G. Bartlett P. F. Turnley A. M. (2004). Axonal regeneration and lack of astrocytic gliosis in EphA4-deficient mice. J. Neurosci. 24, 10064–10073. doi: 10.1523/JNEUROSCI.2981-04.2004, PMID: 15537875 PMC6730186

[ref29] Hammarlund M. Nix P. Hauth L. Jorgensen E. M. Bastiani M. (2009). Axon regeneration requires a conserved MAP kinase pathway. Science 323, 802–806. doi: 10.1126/science.1165527, PMID: 19164707 PMC2729122

[ref30] Hara M. Kobayakawa K. Ohkawa Y. Kumamaru H. Yokota K. Saito T.. (2017). Interaction of reactive astrocytes with type I collagen induces astrocytic scar formation through the integrin–N-cadherin pathway after spinal cord injury. Nat. Med. 23, 818–828. doi: 10.1038/nm.4354, PMID: 28628111

[ref31] Hassan-Montero Y. De-Moya-Anegón F. Guerrero-Bote V. P. (2022). SCImago Graphica: a new tool for exploring and visually communicating data. EPI 8:e310502. doi: 10.3145/epi.2022.sep.02

[ref32] Hellal F. Hurtado A. Ruschel J. Flynn K. C. Laskowski C. J. Umlauf M.. (2011). Microtubule stabilization reduces scarring and causes axon regeneration after spinal cord injury. Science 331, 928–931. doi: 10.1126/science.1201148, PMID: 21273450 PMC3330754

[ref33] Höke A. Redett R. Hameed H. Jari R. Zhou C. Li Z. B.. (2006). Schwann cells express motor and sensory phenotypes that regulate axon regeneration. J. Neurosci. 26, 9646–9655. doi: 10.1523/JNEUROSCI.1620-06.2006, PMID: 16988035 PMC6674436

[ref34] Huang J. K. Jarjour A. A. Nait Oumesmar B. Kerninon C. Williams A. Krezel W.. (2011). Retinoid X receptor gamma signaling accelerates CNS remyelination. Nat. Neurosci. 14, 45–53. doi: 10.1038/nn.2702, PMID: 21131950 PMC4013508

[ref35] Hudson T. W. Liu S. Y. Schmidt C. E. (2004). Engineering an improved acellular nerve graft via optimized chemical processing. Tissue Eng. 10, 1346–1358. doi: 10.1089/1076327042500319, PMID: 15588395

[ref36] Jung H. Yoon B. C. Holt C. E. (2012). Axonal mRNA localization and local protein synthesis in nervous system assembly, maintenance and repair. Nat. Rev. Neurosci. 13, 308–324. doi: 10.1038/nrn3210, PMID: 22498899 PMC3682205

[ref37] Kaneko S. Iwanami A. Nakamura M. Kishino A. Kikuchi K.. (2006). A selective Sema3A inhibitor enhances regenerative responses and functional recovery of the injured spinal cord. Nat. Med. 12, 1380–1389. doi: 10.1038/nm1505, PMID: 17099709

[ref38] Karimi-Abdolrezaee S. Eftekharpour E. Wang J. Morshead C. M. Fehlings M. G. (2006). Delayed transplantation of adult neural precursor cells promotes Remyelination and functional neurological recovery after spinal cord injury. J. Neurosci. 26, 3377–3389. doi: 10.1523/JNEUROSCI.4184-05.2006, PMID: 16571744 PMC6673854

[ref39] Karimi-Abdolrezaee S. Eftekharpour E. Wang J. Schut D. Fehlings M. G. (2010). Synergistic effects of transplanted adult neural stem/progenitor cells, Chondroitinase, and growth factors promote functional repair and plasticity of the chronically injured spinal cord. J. Neurosci. 30, 1657–1676. doi: 10.1523/JNEUROSCI.3111-09.2010, PMID: 20130176 PMC6634011

[ref40] Kerschensteiner M. Schwab M. E. Lichtman J. W. Misgeld T. (2005). In vivo imaging of axonal degeneration and regeneration in the injured spinal cord. Nat. Med. 11, 572–577. doi: 10.1038/nm1229 15821747

[ref41] Kigerl K. Gensel J. Ankeny D. Alexander J. Donnelly D. Popovich P. (2009). Identification of two distinct macrophage subsets with divergent effects causing either neurotoxicity or regeneration in the injured mouse spinal cord. J. Neurosci. 29, 13435–13444. doi: 10.1523/JNEUROSCI.3257-09.2009, PMID: 19864556 PMC2788152

[ref42] Kim J. E. Li S. GrandPré T. Qiu D. Strittmatter S. M. (2003). Axon regeneration in young adult mice lacking Nogo-a/B. Neuron 38, 187–199. doi: 10.1016/S0896-6273(03)00147-8, PMID: 12718854

[ref43] Kim J. E. Liu B. P. Park J. H. Strittmatter S. M. (2004). Nogo-66 receptor prevents Raphespinal and Rubrospinal axon regeneration and limits functional recovery from spinal cord injury. Neuron 44, 439–451. doi: 10.1016/j.neuron.2004.10.015, PMID: 15504325

[ref44] Kingham P. J. Kalbermatten D. F. Mahay D. Armstrong S. J. Wiberg M. Terenghi G. (2007). Adipose-derived stem cells differentiate into a Schwann cell phenotype and promote neurite outgrowth *in vitro*. Exp. Neurol. 207, 267–274. doi: 10.1016/j.expneurol.2007.06.029, PMID: 17761164

[ref45] Koffler J. Zhu W. Qu X. Platoshyn O. Dulin J. N. Brock J.. (2019). Biomimetic 3D-printed scaffolds for spinal cord injury repair. Nat. Med. 25, 263–269. doi: 10.1038/s41591-018-0296-z, PMID: 30643285 PMC6559945

[ref46] Koh H. S. Yong T. Chan C. K. Ramakrishna S. (2008). Enhancement of neurite outgrowth using nano-structured scaffolds coupled with laminin. Biomaterials 29, 3574–3582. doi: 10.1016/j.biomaterials.2008.05.014 18533251

[ref47] Koprivica V. Cho K. S. Park J. B. Yiu G. Atwal J. Gore B.. (2005). EGFR activation mediates inhibition of axon regeneration by myelin and chondroitin sulfate proteoglycans. Science 310, 106–110. doi: 10.1126/science.1115462, PMID: 16210539

[ref48] Kumar A. Godwin J. W. Gates P. B. Garza-Garcia A. A. Brockes J. P. (2007). Molecular basis for the nerve dependence of limb regeneration in an adult vertebrate. Science 318, 772–777. doi: 10.1126/science.1147710, PMID: 17975060 PMC2696928

[ref49] Lang B. T. Cregg J. M. DePaul M. A.. (2015). Modulation of the proteoglycan receptor PTPσ promotes recovery after spinal cord injury. Nature 518, 404–408. doi: 10.1038/nature13974, PMID: 25470046 PMC4336236

[ref50] Lee J. Y. Bashur C. A. Goldstein A. S. Schmidt C. E. (2009). Polypyrrole-coated electrospun PLGA nanofibers for neural tissue applications. Biomaterials 30, 4325–4335. doi: 10.1016/j.biomaterials.2009.04.042, PMID: 19501901 PMC2713816

[ref51] Lee J. K. Kim J. E. Sivula M. Strittmatter S. M. (2004). Nogo receptor antagonism promotes stroke recovery by enhancing axonal plasticity. J. Neurosci. 24, 6209–6217. doi: 10.1523/JNEUROSCI.1643-04.2004, PMID: 15240813 PMC6729662

[ref52] Lee Y. B. Polio S. Lee W. Dai G. Menon L. Carroll R. S.. (2010). Bio-printing of collagen and VEGF-releasing fibrin gel scaffolds for neural stem cell culture. Exp. Neurol. 223, 645–652. doi: 10.1016/j.expneurol.2010.02.014, PMID: 20211178

[ref53] Lee A. C. Yu V. M. Lowe J. B. Brenner M. J. Hunter D. A. Mackinnon S. E.. (2003). Controlled release of nerve growth factor enhances sciatic nerve regeneration. Exp. Neurol. 184, 295–303. doi: 10.1016/S0014-4886(03)00258-9, PMID: 14637100

[ref54] Li Y. Chen J. Zhang C. L. Wang L. Lu D. Katakowski M.. (2005). Gliosis and brain remodeling after treatment of stroke in rats with marrow stromal cells. Glia 49, 407–417. doi: 10.1002/glia.20126, PMID: 15540231

[ref55] Li S. Liu B. P. Budel S. Li M. Ji B. Walus L.. (2004). Blockade of Nogo-66, myelin-associated glycoprotein, and oligodendrocyte myelin glycoprotein by soluble Nogo-66 receptor promotes axonal sprouting and recovery after spinal injury. J. Neurosci. 24, 10511–10520. doi: 10.1523/JNEUROSCI.2828-04.2004, PMID: 15548666 PMC6730300

[ref56] Li S. Strittmatter S. M. (2003). Delayed systemic Nogo-66 receptor antagonist promotes recovery from spinal cord injury. J. Neurosci. 23, 4219–4227. doi: 10.1523/JNEUROSCI.23-10-04219.2003, PMID: 12764110 PMC6741116

[ref57] Liebscher T. Schnell L. Schnell D. Scholl J. Schneider R. Gullo M.. (2005). Nogo-a antibody improves regeneration and locomotion of spinal cord–injured rats. Ann. Neurol. 58, 706–719. doi: 10.1002/ana.20627, PMID: 16173073

[ref58] Liu K. Lu Y. Lee J. K. Samara R. Willenberg R. Sears-Kraxberger I.. (2010). PTEN deletion enhances the regenerative ability of adult corticospinal neurons. Nat. Neurosci. 13, 1075–1081. doi: 10.1038/nn.2603, PMID: 20694004 PMC2928871

[ref59] Lopez-Verrilli M. A. Picou F. Court F. A. (2013). Schwann cell-derived exosomes enhance axonal regeneration in the peripheral nervous system. Glia 61, 1795–1806. doi: 10.1002/glia.22558, PMID: 24038411

[ref60] Lu P. Jones L. L. Snyder E. Y. Tuszynski M. H. (2003). Neural stem cells constitutively secrete neurotrophic factors and promote extensive host axonal growth after spinal cord injury. Exp. Neurol. 181, 115–129. doi: 10.1016/S0014-4886(03)00037-2 12781986

[ref61] Lu P. Jones L. Tuszynski M. (2005). BDNF-expressing marrow stromal cells support extensive axonal growth at sites of spinal cord injury. Exp. Neurol. 191, 344–360. doi: 10.1016/j.expneurol.2004.09.018, PMID: 15649491

[ref62] Lu P. Wang Y. Graham L. McHale K. Gao M. Wu D.. (2012). Long-distance growth and connectivity of neural stem cells after severe spinal cord injury. Cell 150, 1264–1273. doi: 10.1016/j.cell.2012.08.020, PMID: 22980985 PMC3445432

[ref63] Lu P. Woodruff G. Wang Y. Graham L. Hunt M. Wu D.. (2014). Long-distance axonal growth from human induced pluripotent stem cells after spinal cord injury. Neuron 83, 789–796. doi: 10.1016/j.neuron.2014.07.014, PMID: 25123310 PMC4144679

[ref64] Lu P. Yang H. Jones L. L. Filbin M. T. Tuszynski M. H. (2004). Combinatorial therapy with Neurotrophins and cAMP promotes axonal regeneration beyond sites of spinal cord injury. J. Neurosci. 24, 6402–6409. doi: 10.1523/JNEUROSCI.1492-04.2004, PMID: 15254096 PMC6729552

[ref65] MacDonald J. M. Beach M. G. Porpiglia E. Sheehan A. E. Watts R. J. Freeman M. R. (2006). The Drosophila cell corpse engulfment receptor Draper mediates glial clearance of severed axons. Neuron 50, 869–881. doi: 10.1016/j.neuron.2006.04.028, PMID: 16772169

[ref66] Mead B. Tomarev S. (2017). Bone marrow-derived mesenchymal stem cells-derived exosomes promote survival of retinal ganglion cells through miRNA-dependent mechanisms. Stem Cells Transl. Med. 6, 1273–1285. doi: 10.1002/sctm.16-0428, PMID: 28198592 PMC5442835

[ref67] Menet V. Prieto M. Privat A. Ribotta M. G. Y. (2003). Axonal plasticity and functional recovery after spinal cord injury in mice deficient in both glial fibrillary acidic protein and vimentin genes. Proc. Natl. Acad. Sci. USA 100, 8999–9004. doi: 10.1073/pnas.1533187100, PMID: 12861073 PMC166427

[ref68] Mi S. Lee X. Shao Z. Thill G. Ji B. Relton J.. (2004). LINGO-1 is a component of the Nogo-66 receptor/p75 signaling complex. Nat. Neurosci. 7, 221–228. doi: 10.1038/nn1188, PMID: 14966521

[ref69] Monnier P. P. Sierra A. Schwab J. M. Henke-Fahle S. Mueller B. K. (2003). The rho/ROCK pathway mediates neurite growth-inhibitory activity associated with the chondroitin sulfate proteoglycans of the CNS glial scar. Mol. Cell. Neurosci. 22, 319–330. doi: 10.1016/S1044-7431(02)00035-0 12691734

[ref70] Moore D. L. Blackmore M. G. Hu Y. Kaestner K. H. Bixby J. L. Lemmon V. P.. (2009). KLF family members regulate intrinsic axon regeneration ability. Science 326, 298–301. doi: 10.1126/science.1175737, PMID: 19815778 PMC2882032

[ref71] Neuhuber B. Timothy Himes B. Shumsky J. S. Gallo G. Fischer I. (2005). Axon growth and recovery of function supported by human bone marrow stromal cells in the injured spinal cord exhibit donor variations. Brain Res. 1035, 73–85. doi: 10.1016/j.brainres.2004.11.055, PMID: 15713279

[ref72] Nikulina E. Tidwell J. L. Dai H. N. Bregman B. S. Filbin M. T. (2004). The phosphodiesterase inhibitor rolipram delivered after a spinal cord lesion promotes axonal regeneration and functional recovery. Proc. Natl. Acad. Sci. USA 101, 8786–8790. doi: 10.1073/pnas.0402595101, PMID: 15173585 PMC423273

[ref73] Nori S. Okada Y. Yasuda A. Tsuji O. Takahashi Y. Kobayashi Y.. (2011). Grafted human-induced pluripotent stem-cell–derived neurospheres promote motor functional recovery after spinal cord injury in mice. Proc. Natl. Acad. Sci. USA 108, 16825–16830. doi: 10.1073/pnas.1108077108, PMID: 21949375 PMC3189018

[ref74] Oertle T. van der Haar M. E. Bandtlow C. E. Robeva A. Burfeind P. Buss A.. (2003). Nogo-a inhibits neurite outgrowth and cell spreading with three discrete regions-web of Science. J. Neurosci. 23, 5393–5406. doi: 10.1523/JNEUROSCI.23-13-05393.2003, PMID: 12843238 PMC6741224

[ref75] Okano H. Yamanaka S. (2014). iPS cell technologies: significance and applications to CNS regeneration and disease. Mol. Brain 7:22. doi: 10.1186/1756-6606-7-22, PMID: 24685317 PMC3977688

[ref76] Park K. K. Liu K. Hu Y. Smith P. D. Wang C. Cai B.. (2008). Promoting axon regeneration in the adult CNS by modulation of the PTEN/mTOR pathway. Science 322, 963–966. doi: 10.1126/science.1161566, PMID: 18988856 PMC2652400

[ref77] Park J. B. Yiu G. Kaneko S. Wang J. Chang J. He Z. (2005). A TNF receptor family member, TROY, is a Coreceptor with Nogo receptor in mediating the inhibitory activity of myelin inhibitors. Neuron 45, 345–351. doi: 10.1016/j.neuron.2004.12.040, PMID: 15694321

[ref78] Pearse D. D. Pereira F. C. Marcillo A. E. Bates M. L. Berrocal Y. A. Filbin M. T.. (2004). cAMP and Schwann cells promote axonal growth and functional recovery after spinal cord injury. Nat. Med. 10, 610–616. doi: 10.1038/nm1056, PMID: 15156204

[ref79] Prang P. Muller R. Eljaouhari A. Heckmann K. Kunz W. Weber T.. (2006). The promotion of oriented axonal regrowth in the injured spinal cord by alginate-based anisotropic capillary hydrogels. Biomaterials 27, 3560–3569. doi: 10.1016/j.biomaterials.2006.01.053, PMID: 16500703

[ref80] Raivich G. Bohatschek M. da Costa C. Iwata O. Galiano M. Hristova M.. (2004). The AP-1 transcription factor c-Jun is required for efficient axonal regeneration. Neuron 43, 57–67. doi: 10.1016/j.neuron.2004.06.005, PMID: 15233917

[ref81] Rivieccio M. A. Brochier C. Willis D. E. Walker B. A. D'Annibale M. A. McLaughlin K.. (2009). HDAC6 is a target for protection and regeneration following injury in the nervous system. Proc. Natl. Acad. Sci. USA 106, 19599–19604. doi: 10.1073/pnas.0907935106, PMID: 19884510 PMC2780768

[ref82] Ruschel J. Hellal F. Flynn K. C. Dupraz S. Elliott D. A. Tedeschi A.. (2015). Systemic administration of epothilone B promotes axon regeneration after spinal cord injury. Science 348, 347–352. doi: 10.1126/science.aaa2958, PMID: 25765066 PMC4445125

[ref83] Sakai K. Yamamoto A. Matsubara K. Nakamura S. Naruse M. Yamagata M.. (2011). Human dental pulp-derived stem cells promote locomotor recovery after complete transection of the rat spinal cord by multiple neuro-regenerative mechanisms. J Clin Invest. 122:JCI59251, 80–90. doi: 10.1172/JCI59251, PMID: 22133879 PMC3248299

[ref84] Schnell E. Klinkhammer K. Balzer S. Brook G. Klee D. Dalton P.. (2007). Guidance of glial cell migration and axonal growth on electrospun nanofibers of poly-ε-caprolactone and a collagen/poly-ε-caprolactone blend. Biomaterials 28, 3012–3025. doi: 10.1016/j.biomaterials.2007.03.009, PMID: 17408736

[ref85] Seijffers R. Mills C. D. Woolf C. J. (2007). ATF3 increases the intrinsic growth state of DRG neurons to enhance peripheral nerve regeneration. J. Neurosci. 27, 7911–7920. doi: 10.1523/JNEUROSCI.5313-06.2007, PMID: 17652582 PMC6672733

[ref86] Shao Z. Browning J. L. Lee X. Scott M. L. Shulga-Morskaya S. Allaire N.. (2005). TAJ/TROY, an orphan TNF receptor family member, binds Nogo-66 receptor 1 and regulates axonal regeneration. Neuron 45, 353–359. doi: 10.1016/j.neuron.2004.12.050, PMID: 15694322

[ref87] Shen Y. Tenney A. P. Busch S. A. Horn K. P. Cuascut F. X. Liu K.. (2009). PTPσ is a receptor for chondroitin sulfate proteoglycan, an inhibitor of neural regeneration. Science 326, 592–596. doi: 10.1126/science.1178310, PMID: 19833921 PMC2811318

[ref88] Silver J. Miller J. (2004). Regeneration beyond the glial scar. Nat. Rev. Neurosci. 5, 146–156. doi: 10.1038/nrn1326 14735117

[ref89] Simonen M. Pedersen V. Weinmann O. Schnell L. Buss A. Ledermann B.. (2003). Systemic deletion of the myelin-associated outgrowth inhibitor Nogo-a improves regenerative and plastic responses after spinal cord injury. Neuron 38, 201–211. doi: 10.1016/S0896-6273(03)00226-5, PMID: 12718855

[ref90] Sivasankaran R. Pei J. Wang K. C. Zhang Y. P. Shields C. B. Xu X. M.. (2004). PKC mediates inhibitory effects of myelin and chondroitin sulfate proteoglycans on axonal regeneration. Nat. Neurosci. 7, 261–268. doi: 10.1038/nn1193, PMID: 14770187

[ref91] Smith P. D. Sun F. Park K. K. Cai B. Wang C. Kuwako K.. (2009). SOCS3 deletion promotes optic nerve regeneration in vivo. Neuron 64, 617–623. doi: 10.1016/j.neuron.2009.11.021, PMID: 20005819 PMC2796263

[ref92] Stirling D. P. Khodarahmi K. Liu J. McPhail L. T. McBride C. B. Steeves J. D.. (2004). Minocycline treatment reduces delayed oligodendrocyte death, attenuates axonal dieback, and improves functional outcome after spinal cord injury. J. Neurosci. 24, 2182–2190. doi: 10.1523/JNEUROSCI.5275-03.2004, PMID: 14999069 PMC6730425

[ref93] Sun F. Park K. K. Belin S. Wang D. Lu T. Chen G.. (2011). Sustained axon regeneration induced by co-deletion of PTEN and SOCS3. Nature 480, 372–375. doi: 10.1038/nature10594, PMID: 22056987 PMC3240702

[ref94] Tae K. Y. Haftel V. K. Kumar S. Bellamkonda R. V. (2008). The role of aligned polymer fiber-based constructs in the bridging of long peripheral nerve gaps. Biomaterials 29, 3117–3127. doi: 10.1016/j.biomaterials.2008.03.042, PMID: 18448163 PMC2483242

[ref95] Twiss J. L. Fainzilber M. (2009). Ribosomes in axons--scrounging from the neighbors? Trends Cell Biol. 19, 236–243. doi: 10.1016/j.tcb.2009.02.007 19359177

[ref96] Tysseling-Mattiace V. M. Sahni V. Niece K. L. Birch D. Czeisler C. Fehlings M. G.. (2008). Self-assembling nanofibers inhibit glial scar formation and promote axon elongation after spinal cord injury. J. Neurosci. 28, 3814–3823. doi: 10.1523/JNEUROSCI.0143-08.2008, PMID: 18385339 PMC2752951

[ref97] van Eck N. J. Waltman L. (2010). Software survey: VOSviewer, a computer program for bibliometric mapping. Scientometrics 84, 523–538. doi: 10.1007/s11192-009-0146-3, PMID: 20585380 PMC2883932

[ref98] Verma P. Chierzi S. Codd A. M. Campbell D. S. Meyer R. L. Holt C. E.. (2005). Axonal protein synthesis and degradation are necessary for efficient growth cone regeneration. J. Neurosci. 25, 331–342. doi: 10.1523/JNEUROSCI.3073-04.2005, PMID: 15647476 PMC3687202

[ref100] Wells J. E. A. (2003). Neuroprotection by minocycline facilitates significant recovery from spinal cord injury in mice. Brain 126, 1628–1637. doi: 10.1093/brain/awg178, PMID: 12805103

[ref101] Whitlock E. L. Tuffaha S. H. Luciano J. P. Yan Y. Hunter D. A. Magill C. K.. (2009). Processed allografts and type I collagen conduits for repair of peripheral nerve gaps. Muscle Nerve 39, 787–799. doi: 10.1002/mus.21220, PMID: 19291791

[ref102] Willis D. Li K. W. Zheng J. Q. Chang J. H. Smit A. Kelly T.. (2005). Differential transport and local translation of cytoskeletal, injury-response, and neurodegeneration protein mRNAs in axons. J. Neurosci. 25, 778–791. doi: 10.1523/JNEUROSCI.4235-04.2005, PMID: 15673657 PMC6725618

[ref103] Xin H. Katakowski M. Wang F. Qian J. Y. Liu X. S. Ali M. M.. (2017). MicroRNA-17–92 cluster in exosomes enhance neuroplasticity and functional recovery after stroke in rats. Stroke 48, 747–753. doi: 10.1161/STROKEAHA.116.015204, PMID: 28232590 PMC5330787

[ref104] Xin H. Li Y. Cui Y. Yang J. J. Zhang Z. G. Chopp M. (2013). Systemic Administration of Exosomes Released from mesenchymal stromal cells promote functional recovery and neurovascular plasticity after stroke in rats. J. Cereb. Blood Flow Metab. 33, 1711–1715. doi: 10.1038/jcbfm.2013.152, PMID: 23963371 PMC3824189

[ref105] Xin H. Li Y. Liu Z. Wang X. Shang X. Cui Y.. (2013). MiR-133b promotes neural plasticity and functional recovery after treatment of stroke with multipotent mesenchymal stromal cells in rats via transfer of exosome-enriched extracellular particles. Stem Cells 31, 2737–2746. doi: 10.1002/stem.1409, PMID: 23630198 PMC3788061

[ref106] Yamashita T. Tohyama M. (2003). The p75 receptor acts as a displacement factor that releases rho from rho-GDI. Nat. Neurosci. 6, 461–467. doi: 10.1038/nn1045, PMID: 12692556

[ref107] Yan D. Wu Z. Chisholm A. D. Jin Y. (2009). The DLK-1 kinase promotes mRNA stability and local translation in C. elegans synapses and axon regeneration. Cell 138, 1005–1018. doi: 10.1016/j.cell.2009.06.023, PMID: 19737525 PMC2772821

[ref9001] Yin Y. Cui Q. Li Y. Irwin N. Fischer D. Harvey A. R.. (2003). Macrophage-derived factors stimulate optic nerve regeneration. J Neurosci. 23, 2284–93. doi: 10.1523/JNEUROSCI.23-06-02284, PMID: 12657687 PMC6742044

[ref108] Yin Y. Henzl M. T. Lorber B. Nakazawa T. Thomas T. T. Jiang F.. (2006). Oncomodulin is a macrophage-derived signal for axon regeneration in retinal ganglion cells. Nat. Neurosci. 9, 843–852. doi: 10.1038/nn1701, PMID: 16699509

[ref109] Zheng B. Ho C. Li S. Keirstead H. Steward O. Tessier-Lavigne M. (2003). Lack of enhanced spinal regeneration in Nogo-deficient mice. Neuron 38, 213–224. doi: 10.1016/S0896-6273(03)00225-3, PMID: 12718856

